# MCF2Chem: A manually curated knowledge base of biosynthetic compound production

**DOI:** 10.1186/s13068-023-02419-8

**Published:** 2023-11-04

**Authors:** Pengli Cai, Sheng Liu, Dachuan Zhang, Qian-Nan Hu

**Affiliations:** 1 CAS Key Laboratory of Computational Biology, Shanghai Institute of Nutrition and Health, University of Chinese Academy of Sciences, Chinese Academy of Sciences, Shanghai, 200031 China; 2 Ecological Systems Design, Institute of Environmental Engineering, ETH Zurich, 8093 Zurich, Switzerland

**Keywords:** Synthetic biology, Microbial cell factory, Biochemical product, Production database, Recommendation system

## Abstract

**Background:**

Microbes have been used as cell factories to synthesize various chemical compounds. Recent advances in synthetic biological technologies have accelerated the increase in the number and capacity of microbial cell factories; the variety and number of synthetic compounds produced via these cell factories have also grown substantially. However, no database is available that provides detailed information on the microbial cell factories and the synthesized compounds.

**Results:**

In this study, we established MCF2Chem, a manually curated knowledge base on the production of biosynthetic compounds using microbial cell factories. It contains 8888 items of production records related to 1231 compounds that were synthesizable by 590 microbial cell factories, including the production data of compounds (titer, yield, productivity, and content), strain culture information (culture medium, carbon source/precursor/substrate), fermentation information (mode, vessel, scale, and condition), and other information (e.g., strain modification method). The database contains statistical analyses data of compounds and microbial species. The data statistics of MCF2Chem showed that bacteria accounted for 60% of the species and that “fatty acids”, “terpenoids”, and “shikimates and phenylpropanoids” accounted for the top three chemical products. *Escherichia coli*, *Saccharomyces cerevisiae*, *Yarrowia lipolytica*, and *Corynebacterium glutamicum* synthesized 78% of these chemical compounds. Furthermore, we constructed a system to recommend microbial cell factories suitable for synthesizing target compounds and vice versa by combining MCF2Chem data, additional strain- and compound-related data, the phylogenetic relationships between strains, and compound similarities.

**Conclusions:**

MCF2Chem provides a user-friendly interface for querying, browsing, and visualizing detailed statistical information on microbial cell factories and their synthesizable compounds. It is publicly available at https://mcf.lifesynther.com. This database may serve as a useful resource for synthetic biologists.

**Supplementary Information:**

The online version contains supplementary material available at 10.1186/s13068-023-02419-8.

## Background

Synthetic biology, as the core technology of green manufacturing, has advanced rapidly during the past few decades. It is involved in many aspects of life, such as medicine, energy, food, material, and agriculture [1–4]. As highly suitable chassis cells in synthetic biology, microbes are used as cell factories (i.e., microbial chassis) to produce a variety of bulk chemicals and natural products [1, 5–8]. Among them, *Saccharomyces cerevisiae*, *Escherichia coli*, and *Corynebacterium glutamate* are the species most commonly utilized as microbial cell factories and producing a large amount of compounds. However, these model microbial cell factories are insufficient to meet all production targets, largely owing to inherent defects and bottlenecks in the model microbial chassis themselves and the increasing demand for synthetic compounds [9, 10].

With the rapid development of synthetic biological techniques, such as DNA sequencing and CRISPR/Cas technology, more microbes are being engineered for the biosynthesis of various compounds [11]. As of June 2020, the genomes of 11.4% of fungi, 62.8% of bacteria, 69.0% of archaea, and 9.6% of algae have been sequenced, and the CRISPR/Cas gene-editing system has been developed for 157 strains [11]. Technological advances and bottleneck breakthroughs have facilitated the development of microbial cell factories used for biosynthesis [12–14]. Furthermore, the synthetic capacity of microbial cell factories and variety and yield of synthetic compounds produced are constantly improving via metabolic modifications of the microbial chassis in conjunction with fermentation or conversion processes, such as microbial chassis engineering, precursor and cofactor support, competitive pathway blocking, cytotoxicity engineering, and microbial chassis evolution [15–17].

Meanwhile, a number of related tools and databases have been developed for various aspects of microbial biosynthesis [18]. However, to the best of our knowledge, no database is available providing detailed information (i.e., titer, yield, productivity, strain culture, and fermentation condition) regarding microbial cell factories and the compounds biosynthesized by them. Although there are species and compound association databases, such as Cell2Chem and Natural Product Activity and Species Source [19, 20], the relationship between species and compounds is not certain to be a synthetic or production relationship [20], or these databases simply encompass the microbial origin relationship of the compounds [19]. To meet the need for detailed information on compounds biosynthesized by microbial cell factories, Oyetunde et al. manually extracted data from ~ 100 articles and curated a dataset comprising ~ 1200 experimentally implemented cell factories that produced > 20 compounds, mostly focusing on *E. coli* for the production of small molecules [21]. However, this dataset does not include data regarding the biosynthesis of compounds by other microbial cell factories.

Accordingly, the present study established MCF2Chem (https://mcf.lifesynther.com/), a manually curated knowledge base of microbial cell factory biosynthetic compound production. MCF2Chem contains information on microbial cell factories and their biosynthetic compounds extracted from recent synthetic biology reviews, including the information on microbial species, strain culture and fermentation, compounds, and the production data of compounds. Moreover, we also provided statistics for every microbial chassis and compound to facilitate comparison, and a recommendation system to recommend microbial cell factories most suitable for synthesizing target compounds and predict synthesizable compounds by target strains. Thus, this database may serve as a useful resource for synthetic biologists.

## Results

### Database overview

MCF2Chem is the first manually curated knowledge base that details the production of biosynthetic compounds by microbial cell factory and incorporates recommendation system. MCF2Chem includes information on microbial species and the compounds synthesized by those species, production data of the synthesized compounds (titer, yield, productivity, and content), strain culture conditions (carbon source/precursor/substrate, and medium), fermentation information (fermentation mode, vessel, scale, and condition), and other information (e.g., strain modifications). In addition, statistical analyses related to every microbial chassis and compound were automatically performed and presented on the webpage; the recommendation system was built based on data contained in MCF2Chem and additional chemical- and strain-related data. The search function of MCF2Chem allows the required references to be quickly located by querying production data, such as titer, yield, and productivity. Statistical analyses not only provide a general overview of the biosynthesis in microbial cell factories but may also be beneficial for evaluating biosynthesis capacity of target microbial chassis and the biosynthesis situation of target compounds. It is also useful for mining potential chassis for target compounds or potential synthesizable compounds for target chassis.

Data in MCF2Chem were extracted from reviews of metabolic engineering in synthetic biology over the past 5 years (Additional file 1: Table S1). The top three journals contributing the most reviews used for data extraction were “*Applied Microbiology and Biotechnology*”, “*World Journal of Microbiology & Biotechnology*”, *and* “*Biotechnology Advances*” (Additional file 2: Fig. S1). In total, 8888 items of production records were extracted from 268 review articles, involving information from 4765 original microbial metabolic engineering articles (92 records were those of patents; Table 1). The 4765 articles concerned spanned the period from 1946 to 2022, peaking during the 2013–2020 period (Additional file 2: Fig. S2). Many of these articles were published in various new journals devoted to synthetic biology or metabolic engineering, such as “*Metabolic Engineering*”, “*Bioresource Technology*”, “*Microbial Cell Factories*”, and “*Biotechnology for Biofuels*”, which accounted for nearly half of the top 10 source journals (Additional file 2: Fig. S3).

**Table 1:** Statistics of microbial cell factory information in MCF2Chem

Category	Count	Product	Species/others	Article	Review
Bacteria	5276	835	356	2978	195
Yeasts	2585	457	88	1218	136
Fungi	347	47	74	208	32
Microalgae	367	78	66	227	39
Archaea	10	8	6	8	6
Mixed strains	176	69	69	109	38
Other	54	31	35	38	8
None	73	2	0	62	2
Total ^a^	8585	1196	590	4597	266
Total	8888	1231	694	4765	268

Total ^a^: summary of data for all single strains (Bacteria, Yeasts, Fungi, Microalgae, and Archaea)

### Microbial cell factory statistics

**Fig. 1:**
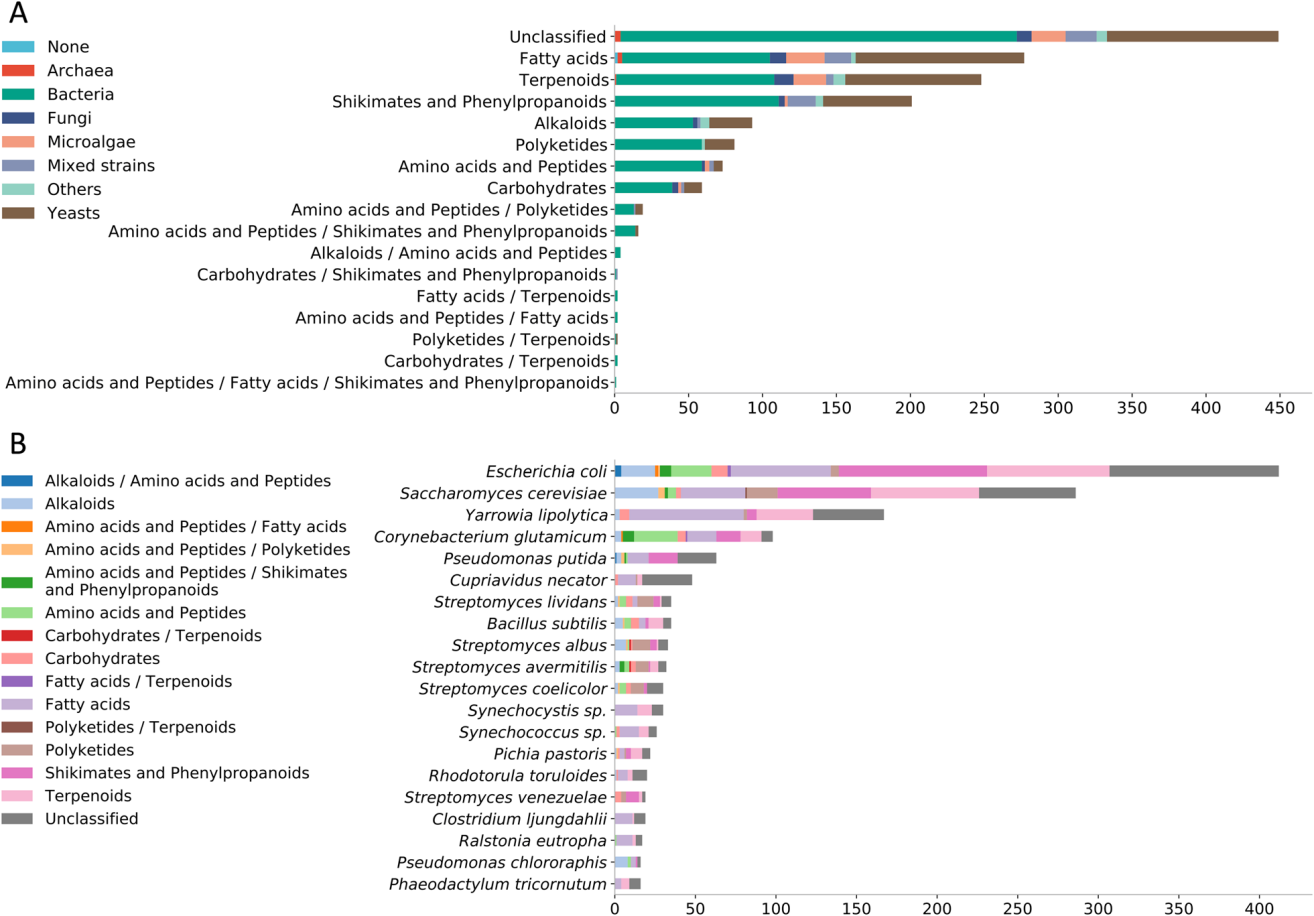
Statistics of microbial chassis strains and their biosynthesized chemical products in MCF2Chem. **A** Category distribution of biosynthesized compounds of different strain categories. **B** Classification of chemical products produced by the top 20 microbial species with the most products. The nc_pathway classification predicted by NPClassifier [22] was used to classify the chemical compound products

MCF2Chem contains data relating to 1231 chemical compound products and 590 microbial species (Table 1). Bacteria were the main producers, both in terms of the number of microbial species used for biosynthesis and types of synthesized compounds. Bacteria accounted for more than 60% of the total microbial chassis and synthesized approximately 68% of the chemical products. Yeasts produced 37% of the chemical products. Fungi and microalgae were similar in most respects, except those microalgae outnumbered fungi in the number of products. In addition to single-strain production, the database covers the production of a small number of mixed strains and other modes of production (Table 1). In terms of the types of compounds synthesized, bacteria and yeast showed similar synthetic profiles. For product quantity, bacteria produced similar quantities of “shikimic acids and phenylpropionic acids”, “terpenoids”, and “fatty acids”, while yeasts were dominant in the production of “fatty acids”, “terpenoids”, and “shikimates and phenylpropyl esters” in that order. The types of compounds synthesized by fungi and microalgae were similar, primarily comprising “fatty acids” and “terpenoids” (Fig. 1A).

In the top 20 microbial species with the most products, *E. coli*, *S. cerevisiae*, *Y. lipolytica*, and *C. glutamate* synthesized ~ 78% of the chemical compounds and were adept at synthesizing “shikimates and phenylpropanoids”, “terpenoids”, “fatty acids”, and “amino acids and peptides”, respectively. Among them, *E. coli* produced a quarter of these compounds (Fig. 1B). *E. coli* and *S. cerevisiae* produced similar types of compounds. *Streptomyces* were adept at synthesizing “polyketides”. *Synechocystis* sp. and *Synechococcus* sp., the microalgae with the most chemical products, mainly synthesized “fatty acids” and “terpenoids” (Fig. 1B).

**Fig. 2:**
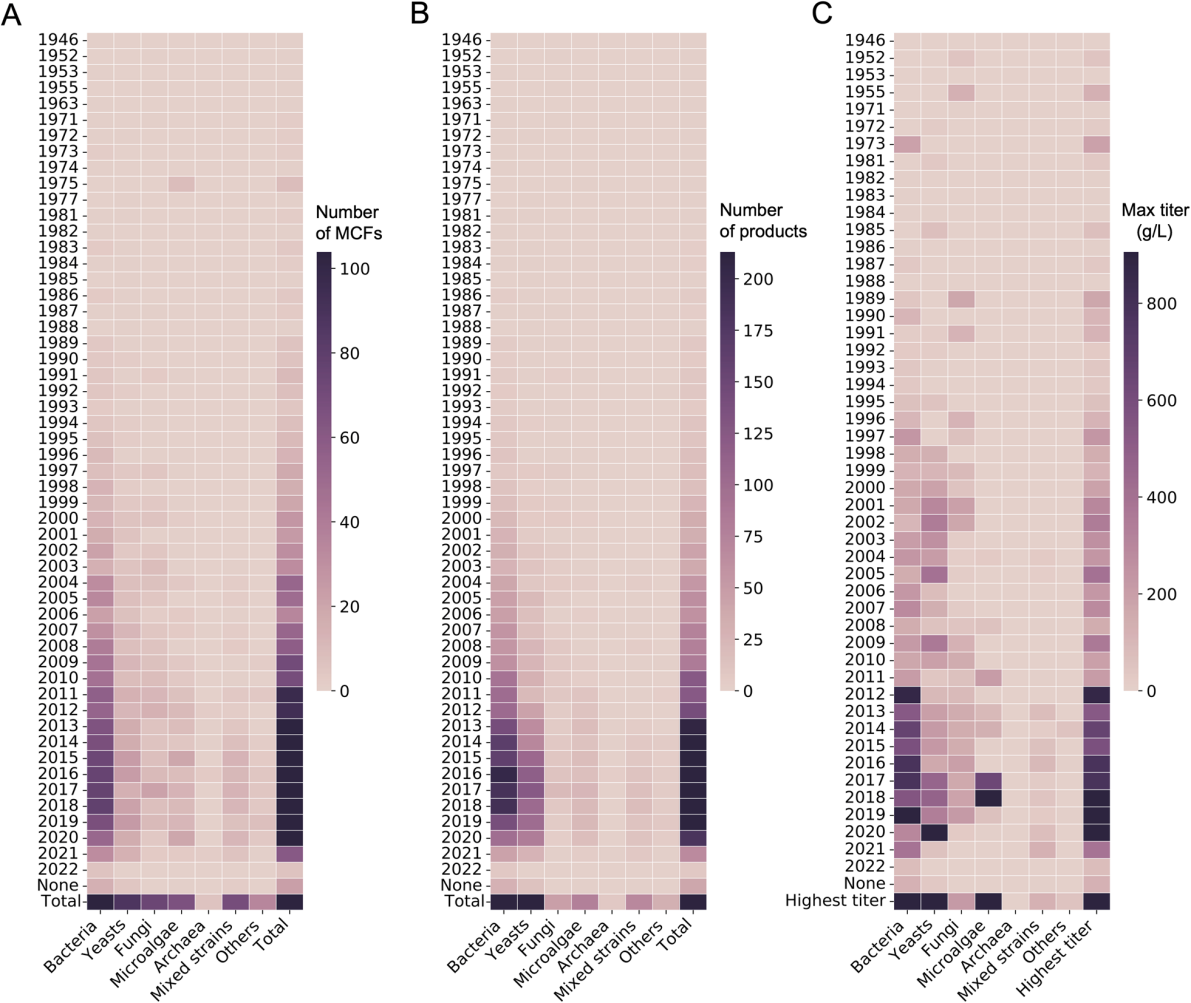
Timeline depicting the number of microbial species, chemical products, and highest titer per strain category. Timelines depicting the development of the **A** number of microbial cell factories (MCFs), **B** number of microbial cell factory products, and **C** highest titer of microbial cell factory products

In terms of temporal development, the number of microbial chassis (especially bacteria) used to synthesize compounds has increased rapidly over the past 20 years. Over the past 10 years, the capability of microalgae to act as microbial cell factories has developed relatively quickly. In addition to the use of single strains, the use of mixed-strain fermentation has gradually increased over this period as well (Fig. 2A). The number and highest titers of compounds, especially those produced by bacteria and yeast, were also improved markedly (Fig. 2B, C). The average titer of compounds synthesized by yeast was lower than that of compounds synthesized by bacteria, which may be due to the increased synthesis proportion of natural products that generally have lower titers (Additional file 2: Fig. S4).

### Chemical compound product statistics

**Fig. 3:**
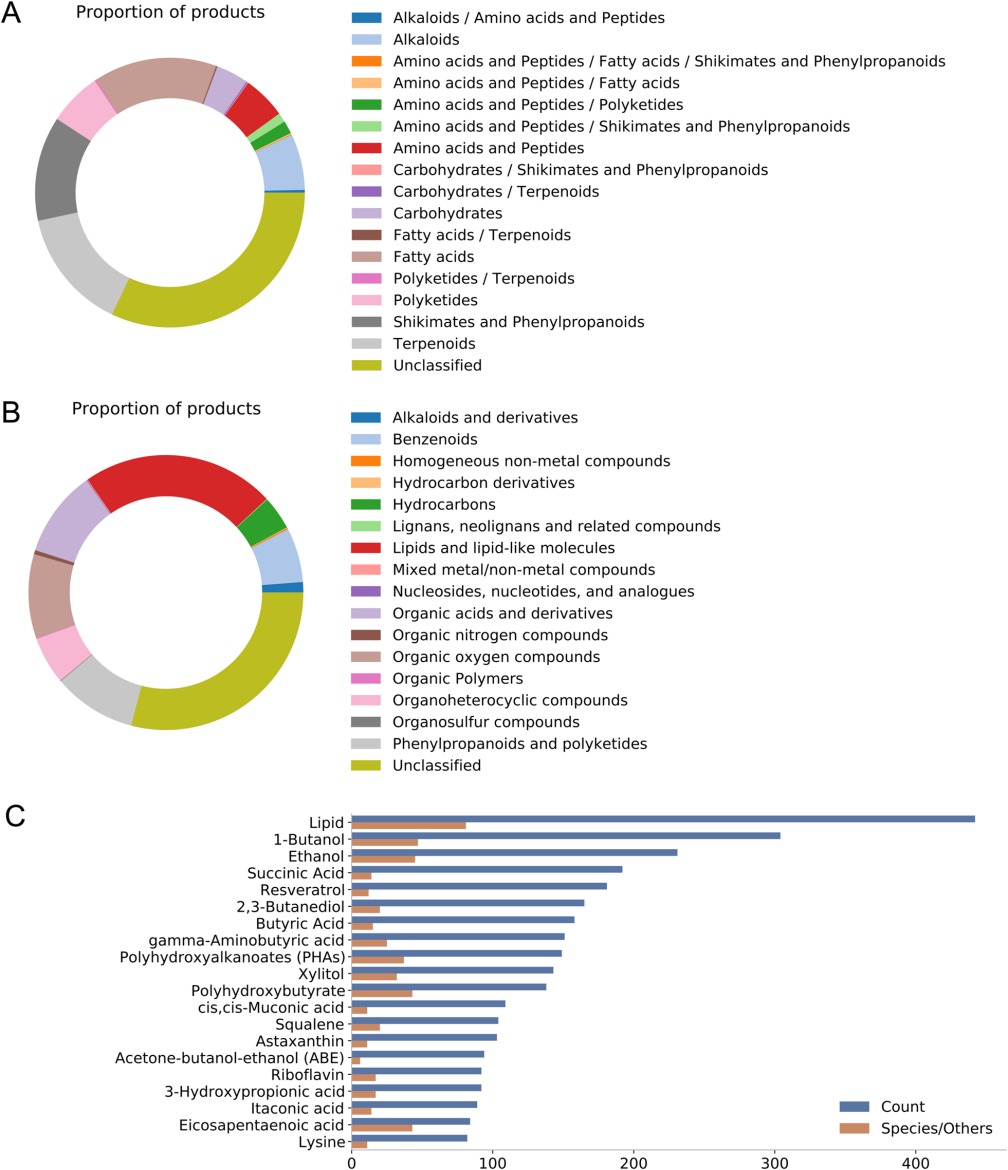
Statistics of chemical compounds synthesized using microbial strains in MCF2Chem. Compound classification results of **A** nc_pathway predicted by NPClassifier and **B** cf_superclass predicted by ClassyFire. **C** Top 20 chemical compounds with the highest count

**Fig. 4:**
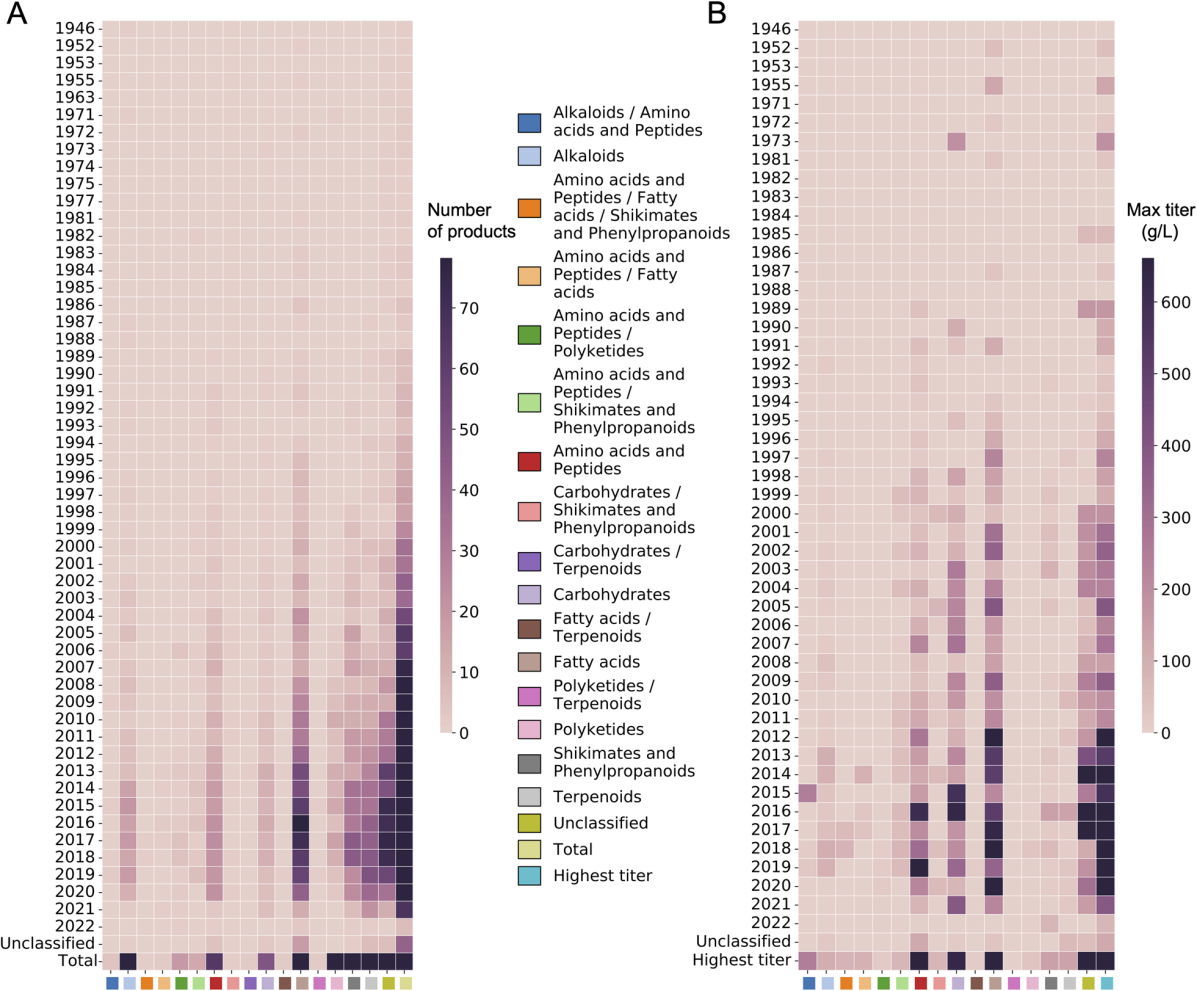
Timeline of the number of chemical products and correlated maximum titers per nc_pathway classification category. The temporal development trend in the microbial biosynthesis of every compound category: **A** number of chemical products and **B** maximum titer

MCF2Chem contains 1231 non-duplicate chemical compound products after data processing. Among them, 835 compounds with chemical structures were involved in the *nc_pathway* classification predicted by NPClassifier [22]. The main compounds synthesized by microbial species were “fatty acids”, “terpenoids”, and “shikimates and phenylpropanoids” (Figs. 3A, 4A). The *cf_superclass* classification predicted by ClassyFire [23] for these compounds indicated that the top three categories of products were “lipids and lipid-like molecules”, “organic acids and derivatives”, and “organic oxygen compounds” (Fig. 3B). The top 10 compound products with the highest counts were lipids, 1-butanol, ethanol, succinic acid, resveratrol, 2,3-butanediol, butyric acid, gamma-aminobutyric acid, polyhydroxyalkanoates, and xylitol (Fig. 3C). The top three compounds with the highest counts in different broad categories were 1-butanol, ethanol, and succinic acid in the “fatty acids” category; squalene, astaxanthin, and lycopene in the “terpenoids” category; resveratrol, shikimic acid, and naringenin in the “shikimates and phenylpropanoids” category; xylitol, mannitol, and fructosylated chondroitin in the “carbohydrates” category; gamma-aminobutyric acid, lysine, and valine in the “amino acids and peptides” category; and riboflavin, violacein, and cadaverine in the “alkaloids” category.

Compounds in the “fatty acids”, “amino acids and peptides”, and “carbohydrates” categories performed well in terms of maximum and average titers (Fig. 4B, Additional file 2: Fig. S5), whereas the product titers of “terpenoids”, “shikimates and phenylpropanoids”, “alkaloids”, and “polyketides” were relatively low. These natural products are secondary metabolites, some of them having very complex structures and low titers, which may explain the generally low average titers of compounds produced by terpene-producing microbial yeasts.

Platform chemicals, including sugar alcohols, furanic compounds, and carboxylic acids, are small molecules that may be synthesized from biomass via chemical conversion or fermentation [24]. The biosyntheses of some common platform chemicals [15, 24–26] were also statistically analyzed (Table 2).

**Table 2:** Statistics of common platform chemicals synthesized using microbial strains in MCF2Chem

Platform chemical	Highest titer (g L^−1^)	Average titer (g L^−1^)	Record
Citric acid	200.0	93.2	54
D-Lactic acid	264.0	91.4	44
L-Lactic acid	221.0	110.4	30
Lactic acid	205.7	52.4	68
Itaconic acid	220.0	39.7	87
Succinic acid	209.7	53.4	185
Propionic acid	135.0	53.5	9
Butyric acid	86.9	28.8	157
L-malic acid	196.0	67.6	21
Fumaric acid	66.3	15.3	13
Ethanol	119.0	22.0	176
Glycerol	130.0	29.5	13
Isoprene	60.0	5.8	66
3-Hydroxypropionic acid	154.0	38.6	91
Xylitol	260.0	50.1	128
Erythritol	243.0	113.1	52
Putrescine	42.3	10.6	16
Cadaverine	103.8	59.0	18
Gamma-aminobutyric acid	2771.0	52.0	147

### Fermentation-related data statistics

MCF2Chem contains 5873 carbon source/substrate/precursor records. Among these, records containing glucose, glycerol, and xylose accounted for 41%, 11%, and 11% of the total records, respectively. CO_2_ and methanol were promising carbon sources, accounting for 2.5% of the records. The top three products that yielded the highest titers when using methanol as a carbon source/substrate/precursor were glutamic acid (60 g L^−1^), polyhydroxybutyrate (52.9 g L^−1^), and poly(3-hydroxybutyrate) (46.1 g L^−1^), which were synthesized by *Bacillus methanolicus*, *Methylorubrum extorquens*, and *Methylobacterium extorquens,* respectively, all of which are species that utilize methanol. The top three corresponding products with the highest titers, using CO_2_ as a carbon source/substrate/precursor, were acetate (59.2 g L^−1^), 2,3-butanediol (32 g L^−1^), and ethanol (20.7 g L^−1^) synthesized by *Acetobacterium woodii*, *Cupriavidus necator,* and *Clostridium ljungdahlii*, respectively, indicating the advantages conferred by these rather than other strains when utilizing different carbon sources.

MCF2Chem also contains 2678 records of fermentation vessels. Notably, different flasks were the main vessels, accounting for 56%, followed by fermenters and reactors, accounting for 33%. The volumes of the fermenters and reactors were typically within 5 L.

### Recommendation system and user interface

Two recommendation function modules were constructed based on evolutionary phylogenetic relationships of strains and compound similarity using MCF2Chem and other auxiliary data to explore potential compounds and chassis. Each module had three recommended routes: S2C/C2S (Strain to Compounds or Compound to Strains), S2C2C/C2S2S, and S2S2C/C2C2S. Diverse recommendation routes provided greater scalability and potential. Users may gain new insights into unreported chemical production or microbial chassis utilization. The compounds or species resulting from the use of different recommended routes were ranked using a corresponding scoring function, which assigned a certain weight to different data for comprehensive consideration. This recommendation system has now been integrated into MCF2Chem.

**Fig. 5:**
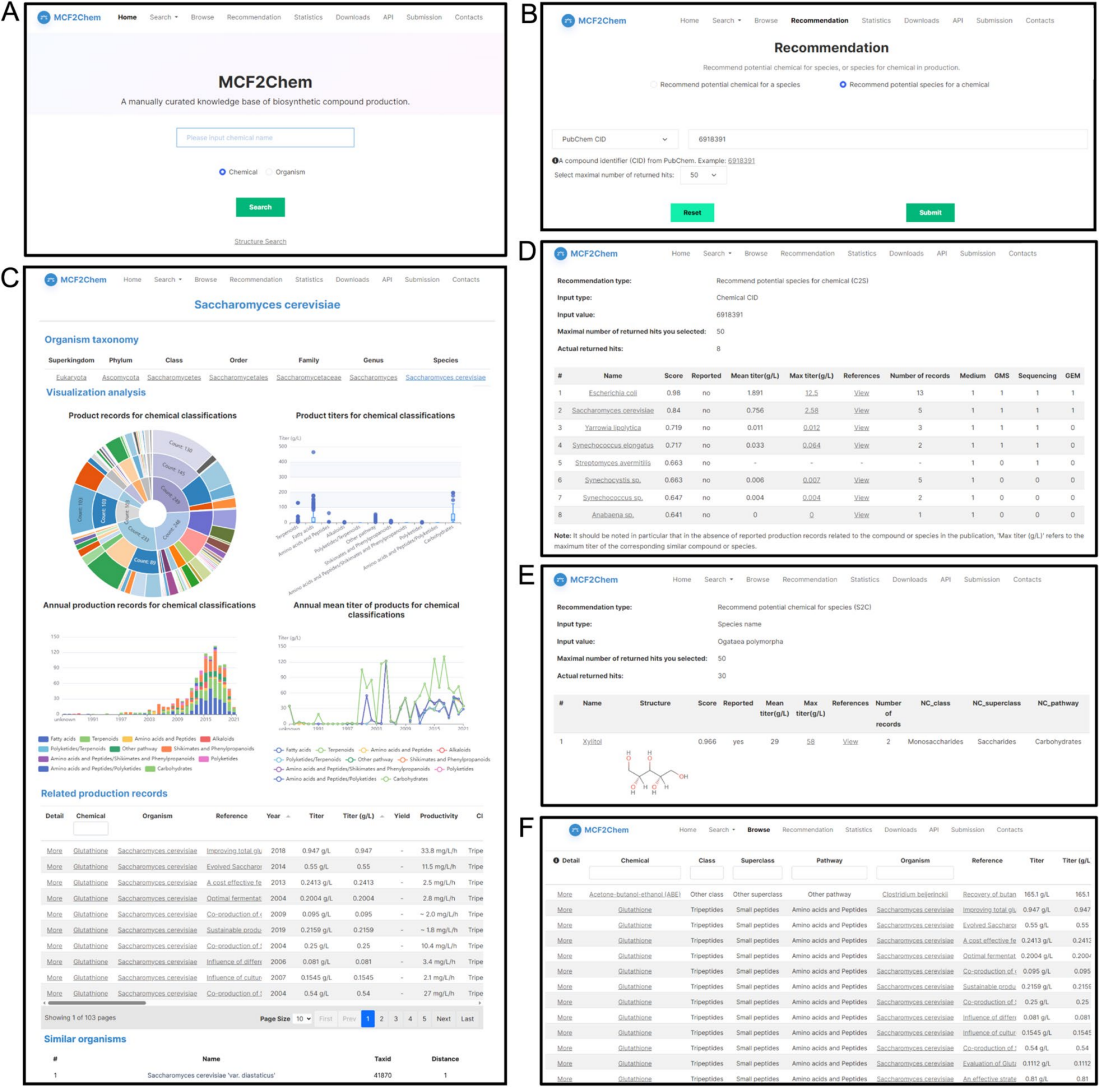
User interface of MCF2Chem. **A** Search home page; **B** recommendation system page; **C** detailed interface of retrieved species with statistical analysis; **D**, **E** recommendation results of retrieved species and compounds; and **F** browser page

MCF2Chem provides retrieval and recommendation pages (Fig. 5A, B). For retrieval, it offers both simple and advanced methods. Compound- and strain-detailed information, including basic information, organism taxonomy, statistics corresponding to all detailed records, and similar compounds or species, can be found on the species and compound *Details* pages (Fig. 5C). The *Recommendation Result* pages of compounds and strains display the corresponding detailed recommendation record, score, and indicate whether the data have been reported (Fig. 5D, E). MCF2Chem also provides a *Browsing* page that presents records of all data including the following: species information and its category; chemical product and its category; production data (titer, yield, productivity, and content); culture and fermentation data (carbon source/precursor/substrate, medium, mode, vessel, scale, and condition); and other data (such as metabolic engineering strategy and strain genotype) (Fig. 5F). Each production record is also available on the *Production Record Details* page. A channel that enables users to upload data to compensate for missing data can also be found in MCF2Chem.

## Discussion

With the increasing demand for green biomanufacturing and the rapid development of corresponding technologies in synthetic biology, the number of microorganisms used for biosynthesis has gradually expanded, and their biosynthetic capacity has also been improved, leading to an increase in the number and production of compounds produced. In this study, we constructed MCF2Chem, a database of the production of microbial biosynthetic compounds. Statistical analyses corresponding to the data presented and simple recommendations for potential chassis and compounds were also incorporated into MCF2Chem.

It is difficult to accurately conduct text mining owing to the complexity of the relationship between various entities of microbial biosynthetic data. Furthermore, manually extracting information directly from original literature is both time-consuming and labor-intensive. Many review articles have periodically summarized and described the categories and yields of the compounds biosynthesized by various microbial cell factories or provided the modification and fermentation information of the microbial cell factories used for biosynthesis of a specific compound or class of compounds [27–32]. Therefore, the data in MCF2Chem were extracted from reviews that covered compounds biosynthesized via microbial strains within the last 5 years, including microbial species, the compounds synthesized using them, related production data, culture conditions, fermentation data, strain modifications, and other information.

MCF2Chem does not only provide a search function, but also facilitates data statistics and comparison, particularly data on titers, yields, and productivities, thus leading to an evaluation of the biosynthetic capacity of various strains and production situation of various compounds. Therefore, data standardization and classification are critical for data statistics. During this process, some difficulties were encountered. Because some compounds are newly synthesized chemicals, biopolymers, or mixtures, approximately 32% of the compounds in MCF2Chem cannot be retrieved from PubChem; thus, they cannot be classified in batches, which is inconvenient for data comparison. Moreover, the production units used were diverse, and some units were difficult to unify. Depending on data characteristics and experimental purposes, researchers tend to choose optimal expression methods and units, leading to diversity in units and increasing the difficulty of data comparison.

Microbial biosynthesis has advanced rapidly over the past decade owing to technological developments, as reflected by an increase in both the number and production capacity of microbial cell factories. In MCF2Chem, 1231 compounds had been biosynthesized by 590 microbial species, with bacteria acting as the main producers. The model microbial chassis, *E. coli*, *S. cerevisiae*, *Y. lipolytica*, *C. glutamicum*, and *P. putida*, biosynthesized 83% of the products. Other strains, such as several microalgae species, which have been explored more recently, have also been found to perform well. Moreover, biosynthesis is no longer limited to a single strain. In summary, microbial chassis can be generally divided into three categories: (a) broad biosynthetic profile strains, such as *E. coli* and *S. cerevisiae*, capable of synthesizing a variety of compounds; (b) featured biosynthesis strains capable of synthesizing a relatively specific class of compounds or exhibiting some special characteristics, such as special carbon source utilization (e.g., *Streptomyces* sp. and *P. pastoris*); and (c) microbial species located between the two previously mentioned types of strains, such as *C. glutamicum*. Although the data of yield and productivity were also important, owing to the limitation of data quantity, titers were selected for production evaluation and statistical analyses in the current study. Titers were improved gradually in recent years, but titers of most secondary metabolites were substantially lower than those of primary metabolites.

As of 2022, 73 countries have been involved in the exploration of microbial biosynthesis, according to incomplete statistics from MCF2Chem (Additional file 2: Fig. S6). China, the US, and South Korea are the top three countries associated with the most of research in this field that also contain the largest number of related research institutions. The highest output ratios were observed in Denmark and Switzerland (Additional file 2: Fig. S7). Among all the institutions, Jiangnan University, the Chinese Academy of Sciences, and Tianjin University ranked as the top three in terms of both the articles and products (Additional file 2: Fig. S8). Importantly, compound biosynthesis of microbial cell factory appears to have entered a phase of rapid development in global research (Additional file 2: Fig. S9).

For microbial chassis recommendation, Ding et al. constructed novoPathFinder based on metabolic pathway design [33] and Cai et al. have recommended this from the perspective of gene editing tools, genome sequencing, and culture conditions [11]. In the current study, data from MCF2Chem were further combined with data from SynBioStrainFinder and genomic metabolic network models to make microbial chassis recommendations.

Although reviews provide great convenience for sorting and processing data, owing to their lagging nature, omission of the latest data is inevitable, and information related to strains or compounds that have not been described by reviews may also be missed (Additional file 2: Table S2). To resolve such issues, a data upload channel for database users has been developed, and MCF2Chem will be updated regularly. In addition, text-mining methods that facilitate database construction will be enacted to reduce dependence on manual effort and facilitate automatic updating. Specifically, a text binary classification model will first be built to identify the literature related to microbial biosynthesis compound production. On this basis, a unified extraction model for microbial biosynthesis production information will be trained with prompt-based learning [34] to identify strain, compound, titer, yield, and productivity information from the literature. Finally, the information automatically recognized by the machine will be updated to the MCF2Chem database after manual review.

## Conclusions

MCF2Chem is the first manually curated database of microbial biosynthetic compound production. MCF2Chem not only includes detailed and statistically analyzed information on microbial chassis, their product compounds, and related production and fermentation information, but also provides a microbial chassis and compound recommendation system. MCF2Chem will continue to expand, aiming to serve as an important resource for expanding microbial strain research and application in biomanufacturing by microbiologists and synthetic biologists.

## Methods

### Data collection and processing

The raw data of MCF2Chem were extracted from reviews of microbial biosynthesis over the last 5 years (from August 1, 2017, to July 31, 2022). A list of all microbes was obtained from the National Center for Biotechnology Information (NCBI) [35]. After manually filtering the titles and abstracts, 268 reviews were obtained (Additional file 1: Table S1), and data from these reviews were extracted using SCITE [36] before being manually curated. Based on the reference columns in review tables, direct references to each record were obtained and supplemented programmatically or manually. Subsequently, these data were used to acquire information on common reference-related fields. Species names were re-extracted from microbial strains and classified as fungi, yeast, bacteria, microalgae, archaea, or mixed strains. The ETE3 software [37] was employed to standardize species names and obtain taxonomic information. NCBI Taxonomy identifiers were utilized to establish data linkages. To ensure chemical compound normalization, chemical names were converted to corresponding structures. To enhance downstream analysis outcomes, any Greek symbols present in the compound names were transcribed to plain text. Retrieval of the compound identifier, structure and relevant data was facilitated by querying PubChem using the processed chemical name. Classification of compounds was performed using ClassyFire [23] and NPClassifier [22]. Physicochemical properties and drug-like filters of the compounds were then assessed using RDKit (http://www.rdkit.org). Production data of compounds were divided into four columns: titer, yield, productivity, and content. The titers of the products were standardized as g L^−1^ to the maximum extent possible, and original units were retained for those that could not be converted. A portion of the yield and productivity data were also subjected to simple unit-to-unit processing. For the convenience of subsequent data statistics, titer range data were divided into maximum and minimum titers, while only titer data sharing the g L^−1^ unit were included in titer-related statistical analyses. Culture conditions included medium and carbon source/substrate/precursor, while fermentation data included fermentation mode, vessel, scale, and condition. All other parts included possible strain modification methods, strain genotypes, and other information.

### Recommendation system construction

In addition to the data in MCF2Chem, additional compound- and strain-related data were collected to recommend compound products and chassis strains. All natural products in LOTUS [38] were downloaded and merged with compounds in MCF2Chem for further use as a candidate chemical compound library of recommendation system. The collected strain-related data included information regarding culture media, genome sequencing, genetic operating system from SynBioStrainFinder [11], and genomic metabolic network models from the Biochemical Genetic and Genomic (BiGG) model database [39]. All data were cleaned and used to construct recommendation system.

Two recommendation function modules were constructed to assist with the recommendation of potential production compounds for target species and potential species for target compounds. For the former (strain to compounds [S2C]), three recommendation routes were designed: (a) retrieve reported compounds produced by targeted species directly from MCF2Chem (S2C); (b) use the result of route “a” as input to search for structurally similar compound molecules in the compound candidate library (S2C2C); and (c) retrieve compounds produced by the nearest neighbor species of the target species in MCF2Chem (S2S2C). Similarly, three recommended routes were proposed to recommend potential strains for target compounds (compound to strains [C2S]): (a) retrieve the production species corresponding to the targeted compound from MCF2Chem (C2S); (b) use the result of route “a” as input to search for species with the closest evolutionary distance among all species (C2S2S); and (c) search for species in MCF2Chem that may produce compounds structurally similar to the target compound (C2C2S). After recalling the compounds or species using different recommended routes, corresponding scoring functions (Eqs. 1, 2) were designed to score all recalled compounds or species:

1 $$rc = log_{3} \left( {p + \frac{{w_{1} t + w_{2} n}}{{w_{1} + w_{2} }} + 1} \right),$$

where $rc$ indicates the recommended score of a compound; $t$ is the corresponding normalized titer; $n$ is the normalized production record count; ${w}_{1}$ and ${w}_{2}$ denote different weighting factors; specific values are listed (Additional file 3); and $p$ is the recommendation route score, the calculation of which is described further (Additional file 3):

2 $$rs = log_{3} \left( {p + \frac{{w_{1} t + w_{2} n + w_{3} c + w_{4} g + w_{5} s + w_{6} m}}{{w_{1} + w_{2} + w_{3} + w_{4} + w_{5} + w_{6} }} + 1} \right),$$

where $rs$ indicates the recommended score of a species; $t$ is the corresponding normalized titer; $n$ is the normalized production record count; $c$, $g$, $s$, and $m$ represent the presence or absence of culture media, genetic operating system, genome sequencing, and genomic metabolic network model for one species, respectively (1 if yes, 0 if no); and ${w}_{1}$, ${w}_{2}$, ${w}_{3}$, ${w}_{4}$, ${w}_{5}$, and ${w}_{6}$ denote different weighting factors, the specific values of which are listed (Additional file 3).

In a concrete implementation, ETE3 [37] was used to calculate the distances between species. To improve the efficacy of implementing similarity calculations across a large number of compounds, Mol2vec [40] was employed to generate the representation of molecular substructures, and the efficient similarity search library Faiss [41] was used to perform similarity calculations for the vectors (Eq. 3):

3 $$\cos \theta = \frac{A \cdot B}{{\left| A \right|\left| B \right|}} = \frac{{\mathop \sum \nolimits_{i = 1}^{n} A_{i} \times B_{i} }}{{\sqrt {\mathop \sum \nolimits_{i = 1}^{n} \left( {A_{i} } \right)^{2} } \times \sqrt {\mathop \sum \nolimits_{i = 1}^{n} \left( {B_{i} } \right)^{2} } }},$$

where ${A}_{i}$ and ${B}_{i}$ are the *i*th components of the molecular vectors $A$ and $B$, respectively, and $n$ = 200.

### System design and implementation

The MCF2Chem web server was deployed in Ubuntu 18.04.2 using multiple frameworks, including FastAPI 0.73.0, Vue.js 2.7.14, and Bootstrap 5.2. Visualization in MCF2Chem was based on the JavaScript libraries ECharts 5.3.3 and Tabulator 5.4.2. All data for the project were stored in the flexible NoSQL database MongoDB 5.0.4. The RDKit 2020.09.1.0 (http://www.rdkit.org) was used for chemical similarity searches, and JSME v2022-09-26 [42] was used for molecular structural input.

### Supplementary Information

**Additional file 1: Table S1**. List of reviews used for data extraction.

**Additional file 2: Figure S1**. Top 10 journals contributing the most reviews used for data extraction. **Figure S2**. Time statistics of the original articles, countries, and institutions for microbial cell factory biosynthesis. **Figure S3**. Time statistics of the top 20 journals contributing the most original articles on microbial cell factory biosynthesis. **Figure S4**. Development timeline of the average titer of microbial cell factory biosynthesis. **Figure S5**. Time statistics of the average titer of microbial cell factory biosynthesis in every product category. **Figure S6**. Global distribution of microbial cell factory biosynthetic chemical products. **Figure S7**. Top 10 countries contributing the most data to microbial cell factory biosynthesis. **Figure S8**. Top 10 institutions contributing the most data to microbial cell factory biosynthesis. **Figure S9**. Timeline depicting trends in the development of various aspects of microbial cell factory biosynthesis. **Table S2**. MCF2Chem database coverage statistical analysis using the journal *Metabolic Engineering* as an example.

**Additional file 3.** Scoring functions for chemical and species recommendation.

## Data Availability

All data are available at https://mcf.lifesynther.com.

## Abbreviations

BiGG model: Biochemical Genetic and Genomic model
CRISPR/Cas: Clustered regularly interspaced short palindromic repeats/associated protein
NCBI: National Center for Biotechnology Information

## References

[1] Yuan SF, Alper HS (2019). Metabolic engineering of microbial cell factories for production of nutraceuticals. Microb Cell Fact.

[2] Liu AP, Appel EA, Ashby PD, Baker BM, Franco E, Gu L, Haynes K, Joshi NS, Kloxin AM, Kouwer PHJ (2022). The living interface between synthetic biology and biomaterial design. Nat Mater.

[3] Roell MS, Zurbriggen MD (2020). The impact of synthetic biology for future agriculture and nutrition. Curr Opin Biotechnol.

[4] Brooks SM, Alper HS (2021). Applications, challenges, and needs for employing synthetic biology beyond the lab. Nat Commun.

[5] Cho JS, Kim GB, Eun H, Moon CW, Lee SY (2022). Designing microbial cell factories for the production of chemicals. JACS Au.

[6] Agrawal K, Gupta VK, Verma P (2022). Microbial cell factories a new dimension in bio-nanotechnology: exploring the robustness of nature. Crit Rev Microbiol.

[7] Han X, Liu J, Tian S, Tao F, Xu P (2022). Microbial cell factories for bio-based biodegradable plastics production. iScience..

[8] Murphy CD (2012). The microbial cell factory. Org Biomol Chem.

[9] Liu J, Wang X, Dai G, Zhang Y, Bian X (2022). Microbial chassis engineering drives heterologous production of complex secondary metabolites. Biotechnol Adv.

[10] Eisenstein M (2016). Living factories of the future. Nature.

[11] Cai P, Han M, Zhang R, Ding S, Zhang D, Liu D, Liu S, Hu QN (2022). SynBioStrainFinder: a microbial strain database of manually curated CRISPR/Cas genetic manipulation system information for biomanufacturing. Microb Cell Fact.

[12] Si T, Xiao H, Zhao H (2015). Rapid prototyping of microbial cell factories via genome-scale engineering. Biotechnol Adv.

[13] Leavell MD, Singh AH, Kaufmann-Malaga BB (2020). High-throughput screening for improved microbial cell factories, perspective and promise. Curr Opin Biotechnol.

[14] Jakočiūnas T, Jensen MK, Keasling JD (2016). CRISPR/Cas9 advances engineering of microbial cell factories. Metab Eng.

[15] Son J, Sohn YJ, Baritugo KA, Jo SY, Song HM, Park SJ (2023). Recent advances in microbial production of diamines, aminocarboxylic acids, and diacids as potential platform chemicals and bio-based polyamides monomers. Biotechnol Adv.

[16] Gustavsson M, Lee SY (2016). Prospects of microbial cell factories developed through systems metabolic engineering. Microb Biotechnol.

[17] Ding Q, Ye C (2023). Microbial cell factories based on filamentous bacteria, yeasts, and fungi. Microb Cell Fact.

[18] Otero-Muras I, Carbonell P (2021). Automated engineering of synthetic metabolic pathways for efficient biomanufacturing. Metab Eng.

[19] Zeng X, Zhang P, He W, Qin C, Chen S, Tao L, Wang Y, Tan Y, Gao D, Wang B (2018). NPASS: natural product activity and species source database for natural product research, discovery and tool development. Nucleic Acids Res.

[20] Liu D, Han M, Tian Y, Gong L, Jia C, Cai P, Tu W, Chen J, Hu QN (2021). Cell 2Chem: mining explored and unexplored biosynthetic chemical spaces. Bioinformatics.

[21] Oyetunde T, Liu D, Martin HG, Tang YJ (2019). Machine learning framework for assessment of microbial factory performance. PLoS ONE.

[22] Kim HW, Wang M, Leber CA, Nothias LF, Reher R, Kang KB, van der Hooft JJJ, Dorrestein PC, Gerwick WH, Cottrell GW (2021). NPClassifier: a deep neural network-based structural classification tool for natural products. J Nat Prod.

[23] Djoumbou Feunang Y, Eisner R, Knox C, Chepelev L, Hastings J, Owen G, Fahy E, Steinbeck C, Subramanian S, Bolton E (2016). ClassyFire: automated chemical classification with a comprehensive, computable taxonomy. J Cheminform.

[24] Nakagawa Y, Kasumi T, Ogihara J, Tamura M, Arai T, Tomishige K (2020). Erythritol: Another C4 Platform Chemical in Biomass Refinery. ACS Omega.

[25] Li J, Rong L, Zhao Y, Li S, Zhang C, Xiao D, Foo JL, Yu A (2020). Next-generation metabolic engineering of non-conventional microbial cell factories for carboxylic acid platform chemicals. Biotechnol Adv.

[26] Bozell JJ, Petersen GR (2010). Technology development for the production of biobased products from biorefinery carbohydrates—the US department of energy’s “Top 10” revisited. Green Chem.

[27] Nepal KK, Wang G (2019). Streptomycetes: Surrogate hosts for the genetic manipulation of biosynthetic gene clusters and production of natural products. Biotechnol Adv.

[28] Pontrelli S, Chiu TY, Lan EI, Chen FY, Chang P, Liao JC (2018). *Escherichia coli* as a host for metabolic engineering. Metab Eng.

[29] Choi SY, Rhie MN, Kim HT, Joo JC, Cho IJ, Son J, Jo SY, Sohn YJ, Baritugo KA, Pyo J (2020). Metabolic engineering for the synthesis of polyesters: a 100-year journey from polyhydroxyalkanoates to non-natural microbial polyesters. Metab Eng.

[30] Huccetogullari D, Luo ZW, Lee SY (2019). Metabolic engineering of microorganisms for production of aromatic compounds. Microb Cell Fact.

[31] Tippelt A, Nett M (2021). *Saccharomyces cerevisiae* as host for the recombinant production of polyketides and nonribosomal peptides. Microb Cell Fact.

[32] Abdel-Mawgoud AM, Markham KA, Palmer CM, Liu N, Stephanopoulos G, Alper HS (2018). Metabolic engineering in the host *Yarrowia lipolytica*. Metab Eng.

[33] Ding S, Tian Y, Cai P, Zhang D, Cheng X, Sun D, Yuan L, Chen J, Tu W, Wei DQ, Hu QN (2020). novoPathFinder: a webserver of designing novel-pathway with integrating GEM-model. Nucleic Acids Res.

[34] Lu Y, Liu Q, Dai D, Xiao X, Lin H, Han X, Sun L, Wu H (2022). Unified structure generation for universal information extraction. Annu Meet Assoc Comput Linguist.

[35] Federhen S (2012). The NCBI taxonomy database. Nucleic Acids Res.

[36] Cai P, Liu S, Zhang D, Xing H, Han M, Liu D, Gong L, Hu Q-N (2023). SynBioTools: a one-stop facility for searching and selecting synthetic biology tools. BMC Bioinf.

[37] Huerta-Cepas J, Serra F, Bork P (2016). ETE 3: Reconstruction, analysis, and visualization of phylogenomic data. Mol Biol Evol.

[38] Rutz A, Sorokina M, Galgonek J, Mietchen D, Willighagen E, Gaudry A, Graham JG, Stephan R, Page R, Vondrášek J (2022). The LOTUS initiative for open knowledge management in natural products research. Elife.

[39] King ZA, Lu J, Dräger A, Miller P, Federowicz S, Lerman JA, Ebrahim A, Palsson BO, Lewis NE (2016). BiGG models: a platform for integrating, standardizing and sharing genome-scale models. Nucleic Acids Res.

[40] Jaeger S, Fulle S, Turk S (2018). Mol2vec: unsupervised machine learning approach with chemical intuition. J Chem Inf Model.

[41] Johnson J, Douze M, Jégou H (2021). Billion-scale similarity search with GPUs. IEEE Trans Big Data.

[42] Bienfait B, Ertl P (2013). JSME: a free molecule editor in JavaScript. J Cheminf.

