# Supplementary material for: MCF2Chem: A manually curated knowledge base of biosynthetic compound production
Source: Biotechnol Biofuels Bioprod. 2023 Nov 4;16:167. doi: 10.1186/s13068-023-02419-8 (PMC10625697; doi:10.1186/s13068-023-02419-8)
Supplement: Supplementary file 1 — Additional file 1: Table S1. List of reviews used for data extraction. [file 13068_2023_2419_MOESM1_ESM.docx]

Table S1 List of reviews used for data extraction.

| Review_title | Review_doi |
| --- | --- |
| 2016-Engineering microbes for isoprene production | 10.1016/j.ymben.2016.07.005 |
| 2017-Biological production of l-malate: recent advances and future prospects | 10.1007/s11274-017-2349-8 |
| 2017-Biotechnological production of aromatic compounds of the extended shikimate pathway from renewable biomass | 10.1016/j.jbiotec.2016.11.016 |
| 2017-Co-production of microbial polyhydroxyalkanoates with other chemicals | 10.1016/j.ymben.2017.07.007 |
| 2017-Current advances of succinate biosynthesis in metabolically engineered Escherichia coli | 10.1016/j.biotechadv.2017.09.007 |
| 2017-Current status on metabolic engineering for the production of l-aspartate family amino acids and derivatives | 10.1016/j.biortech.2017.05.145 |
| 2017-Engineering metabolic pathways in Escherichia coli for constructing a “microbial chassis” for biochemical production | 10.1016/j.biortech.2017.05.008 |
| 2017-Engineering Saccharomyces cerevisiae for high-level synthesis of fatty acids and derived products | 10.1093/femsyr/fox071 |
| 2017-Metabolic Engineering of Oleaginous Yeasts for Production of Fuels and Chemicals | 10.3389/fmicb.2017.02185 |
| 2017-Microbial conversion of biomass into bio-based polymers | 10.1016/j.biortech.2017.06.135 |
| 2017-Microbial production of rhamnolipids: opportunities, challenges and strategies | 10.1186/s12934-017-0753-2 |
| 2017-Novel technologies combined with traditional metabolic engineering strategies facilitate the construction of shikimate-producing Escherichia coli | 10.1186/s12934-017-0773-y |
| 2017-Production of anthocyanins in metabolically engineered microorganisms: Current status and perspectives | 10.1016/j.synbio.2017.10.005 |
| 2017-Production of chemicals and proteins using biomass-derived substrates from a Streptomyces host | 10.1016/j.biortech.2017.06.001 |
| 2017-State of the art review of biofuels production from lignocellulose by thermophilic bacteria | 10.1016/j.biortech.2017.05.142 |
| 2017-Synthetic biology for manufacturing chemicals: constraints drive the use of non-conventional microbial platforms | 10.1007/s00253-017-8489-9 |
| 2018-Advances and prospects of Bacillus subtilis cellular factories: From rational design to industrial applications | 10.1016/j.ymben.2018.05.006 |
| 2018-Advances in synthetic biology of oleaginous yeast Yarrowia lipolytica for producing non-native chemicals | 10.1007/s00253-018-9099-x |
| 2018-Biosynthesis of d-lactic acid from lignocellulosic biomass | 10.1007/s10529-018-2588-2 |
| 2018-Biotechnological production of mono- and diamines using bacteria: recent progress, applications, and perspectives | 10.1007/s00253-018-8890-z |
| 2018-Butyric acid: Applications and recent advances in its bioproduction | 10.1016/j.biotechadv.2018.09.005 |
| 2018-Current state and perspectives in hydrogen production by Escherichia coli: roles of hydrogenases in glucose or glycerol metabolism | 10.1007/s00253-018-8752-8 |
| 2018-Current Status of Microbial Phenylethanoid Biosynthesis | 10.4014/jmb.1805.05021 |
| 2018-Engineering strategies for enhanced production of protein and bio-products in Pichia pastoris: A review | 10.1016/j.biotechadv.2017.11.002 |
| 2018-Escherichia coli as a host for metabolic engineering | 10.1016/j.ymben.2018.04.008 |
| 2018-Formation of folates by microorganisms: towards the biotechnological production of this vitamin | 10.1007/s00253-018-9266-0 |
| 2018-Genetic and metabolic engineering for microbial production of poly-γ-glutamic acid | 10.1016/j.biotechadv.2018.05.006 |
| 2018-Heterologous production of resveratrol in bacterial hosts: current status and perspectives | 10.1007/s11274-018-2506-8 |
| 2018-Holistic Approaches in Lipid Production by Yarrowia lipolytica | 10.1016/j.tibtech.2018.06.007 |
| 2018-Lactic acid bacteria: from starter cultures to producers of chemicals | 10.1093/femsle/fny213 |
| 2018-Metabolic engineering in the host Yarrowia lipolytica | 10.1016/j.ymben.2018.07.016 |
| 2018-Metabolic engineering of Corynebacterium glutamicum for fermentative production of chemicals in biorefinery | 10.1007/s00253-018-8896-6 |
| 2018-Metabolic engineering of Escherichia coli for the production of isoprenoids | 10.1093/femsle/fny079 |
| 2018-Metabolic engineering strategies for enhanced shikimate biosynthesis: current scenario and future developments | 10.1007/s00253-018-9222-z |
| 2018-Metabolic regulation in solventogenic clostridia: regulators, mechanisms and engineering | 10.1016/j.biotechadv.2018.02.012 |
| 2018-Microbial conversion of xylose into useful bioproducts | 10.1007/s00253-018-9294-9 |
| 2018-Microbial production of alka(e)ne biofuels | 10.1016/j.copbio.2017.08.009 |
| 2018-Microbial Production of l-Serine from Renewable Feedstocks | 10.1016/j.tibtech.2018.02.001 |
| 2018-Opportunities, challenges, and future perspectives of succinic acid production by Actinobacillus succinogenes | 10.1007/s00253-018-9379-5 |
| 2018-Polyunsaturated fatty acids in marine bacteria and strategies to enhance their production | 10.1007/s00253-018-9063-9 |
| 2018-Potential and limitations of Klebsiella pneumoniae as a microbial cell factory utilizing glycerol as the carbon source | 10.1016/j.biotechadv.2017.10.004 |
| 2018-Process engineering for microbial production of 3-hydroxypropionic acid | 10.1016/j.biotechadv.2018.03.020 |
| 2018-Production of plant-derived polyphenols in microorganisms: current state and perspectives | 10.1007/s00253-018-8747-5 |
| 2018-Production of L-valine from metabolically engineered Corynebacterium glutamicum | 10.1007/s00253-018-8952-2 |
| 2018-Recent advances and strategies in process and strain engineering for the production of butyric acid by microbial fermentation | 10.1016/j.biortech.2018.01.007 |
| 2018-Recent advances in biological production of erythritol | 10.1080/07388551.2017.1380598 |
| 2018-Recent advances in metabolic engineering of Corynebacterium glutamicum for bioproduction of value-added aromatic chemicals and natural products | 10.1007/s00253-018-9289-6 |
| 2018-Recent advances in microbial production of aromatic natural products and their derivatives | 10.1007/s00253-017-8599-4 |
| 2018-Recent advances in microbial production of mannitol: utilization of low-cost substrates, strain development and regulation strategies | 10.1007/s11274-018-2425-8 |
| 2018-Rediscovering Acetate Metabolism: Its Potential Sources and Utilization for Biobased Transformation into Value-Added Chemicals | 10.1021/acs.jafc.8b00458 |
| 2018-Solvent production by engineered Ralstonia eutropha: channeling carbon to biofuel | 10.1007/s00253-018-9026-1 |
| 2018-Terpenoid Metabolic Engineering in Photosynthetic Microorganisms | 10.3390/genes9110520 |
| 2018-The Engineering Potential of Rhodosporidium toruloides as a Workhorse for Biotechnological Applications | 10.1016/j.tibtech.2017.10.013 |
| 2018-Transport and metabolic engineering of the cell factory Corynebacterium glutamicum | 10.1093/femsle/fny166 |
| 2018-Value-added biotransformation of cellulosic sugars by engineered Saccharomyces cerevisiae | 10.1016/j.biortech.2018.04.013 |
| 2018-Xylose transport in yeast for lignocellulosic ethanol production: Current status | 10.1016/j.jbiosc.2017.10.006 |
| 2019-Acetone-free biobutanol production: Past and recent advances in the Isopropanol-Butanol-Ethanol (IBE) fermentation | 10.1016/j.biortech.2019.121425 |
| 2019-Advances in 2-phenylethanol production from engineered microorganisms | 10.1016/j.biotechadv.2019.02.005 |
| 2019-Advances in the metabolic engineering of Yarrowia lipolytica for the production of terpenoids | 10.1016/j.biortech.2019.02.116 |
| 2019-Biobutanol Production from Crystalline Cellulose through Consolidated Bioprocessing | 10.1016/j.tibtech.2018.08.007 |
| 2019-Bio-conversion of methane into high profit margin compounds: an innovative, environmentally friendly and cost-effective platform for methane abatement | 10.1007/s11274-018-2587-4 |
| 2019-Biomass-Derived Production of Itaconic Acid as a Building Block in Specialty Polymers | 10.3390/polym11061035 |
| 2019-Bio-solar cell factories for photosynthetic isoprenoids production | 10.1007/s00425-018-2969-8 |
| 2019-Biosynthesis of polymalic acid in fermentation: advances and prospects for industrial application | 10.1080/07388551.2019.1571008 |
| 2019-Biosynthesis of resveratrol and piceatannol in engineered microbial strains: achievements and perspectives | 10.1007/s00253-019-09672-8 |
| 2019-Biosynthesis of ω-hydroxy fatty acids and related chemicals from natural fatty acids by recombinant Escherichia coli | 10.1007/s00253-018-9503-6 |
| 2019-Biosynthetic strategies to produce xylitol: an economical venture | 10.1007/s00253-019-09881-1 |
| 2019-Biotechnological Advances in Resveratrol Production and its Chemical Diversity | 10.3390/molecules24142571 |
| 2019-Biotechnological production of glycolic acid and ethylene glycol: current state and perspectives | 10.1007/s00253-019-09640-2 |
| 2019-Clostridial whole cell and enzyme systems for hydrogen production: current state and perspectives | 10.1007/s00253-018-9514-3 |
| 2019-Consolidated bioprocessing for butanol production of cellulolytic Clostridia: development and optimization | 10.1111/1751-7915.13478 |
| 2019-Engineering Clostridium for improved solvent production: recent progress and perspective | 10.1007/s00253-019-09916-7 |
| 2019-Engineering Saccharomyces cerevisiae cells for production of fatty acid-derived biofuels and chemicals | 10.1098/rsob.190049 |
| 2019-How to make the reducing power of H2 available for in vivo biosyntheses and biotransformations | 10.1016/j.cbpa.2018.11.020 |
| 2019-Metabolic engineering for improving l-tryptophan production in Escherichia coli | 10.1007/s10295-018-2106-5 |
| 2019-Metabolic engineering for the production of l-phenylalanine in Escherichia coli | 10.1007/s13205-019-1619-6 |
| 2019-Metabolic engineering of microbial cell factories for production of nutraceuticals | 10.1186/s12934-019-1096-y |
| 2019-Metabolic engineering of microorganisms for production of aromatic compounds | 10.1186/s12934-019-1090-4 |
| 2019-Metabolic engineering of microorganisms for the production of ethanol and butanol from oxides of carbon | 10.1007/s00253-019-10072-1 |
| 2019-Microbial cell factories for the sustainable manufacturing of B vitamins | 10.1016/j.copbio.2018.07.006 |
| 2019-Microbial production of 2,3-butanediol for industrial applications | 10.1007/s10295-019-02231-0 |
| 2019-Microbial production of butyl butyrate, a flavor and fragrance compound | 10.1007/s00253-018-09603-z |
| 2019-Oleaginous yeast for biofuel and oleochemical production | 10.1016/j.copbio.2019.02.011 |
| 2019-Organic Wastes as Feedstocks for Non-Conventional Yeast-Based Bioprocesses | 10.3390/microorganisms7080229 |
| 2019-Outline of the biosynthesis and regulation of ergosterol in yeast | 10.1007/s11274-019-2673-2 |
| 2019-Production of 3-Hydroxypropanoic Acid From Glycerol by Metabolically Engineered Bacteria | 10.3389/fbioe.2019.00124 |
| 2019-Production of butanol from biomass: recent advances and future prospects | 10.1007/s11356-019-05437-y |
| 2019-Production of fuels and chemicals from renewable resources using engineered Escherichia coli | 10.1016/j.biotechadv.2019.06.001 |
| 2019-Production of plant metabolites with applications in the food industry using engineered microorganisms | 10.1016/j.copbio.2018.07.008 |
| 2019-Recent advancement of engineering microbial hosts for the biotechnological production of flavonoids | 10.1007/s11033-019-05066-1 |
| 2019-Recent advancements in fungal-derived fuel and chemical production and commercialization | 10.1016/j.copbio.2018.08.014 |
| 2019-Recent advances in engineering Corynebacterium glutamicum for utilization of hemicellulosic biomass | 10.1016/j.copbio.2018.11.004 |
| 2019-Recent advances in lignin valorization with bacterial cultures: microorganisms, metabolic pathways, and bio-products | 10.1186/s13068-019-1376-0 |
| 2019-Recent Advances of L-ornithine Biosynthesis in Metabolically Engineered Corynebacterium glutamicum | 10.3389/fbioe.2019.00440 |
| 2019-Strain engineering for microbial production of value-added chemicals and fuels from glycerol | 10.1016/j.biotechadv.2018.10.006 |
| 2019-Strategy for improving L-isoleucine production efficiency in Corynebacterium glutamicum | 10.1007/s00253-019-09632-2 |
| 2019-Streptomycetes: Surrogate Hosts for the Genetic Manipulation of Biosynthetic Gene Clusters and Production of Natural Products | 10.1016/j.biotechadv.2018.10.003 |
| 2019-Systems metabolic engineering for citric acid production by Aspergillus niger in the post-genomic era | 10.1186/s12934-019-1064-6 |
| 2019-Yarrowia lipolytica: more than an oleaginous workhorse | 10.1007/s00253-019-10200-x |
| 2020-100 Years Later, What Is New in Glycerol Bioproduction? | 10.1016/j.tibtech.2020.02.001 |
| 2020-Advances in sophorolipid-producing strain performance improvement and fermentation optimization technology | 10.1007/s00253-020-10964-7 |
| 2020-An Overview of Potential Oleaginous Microorganisms and Their Role in Biodiesel and Omega-3 Fatty Acid-Based Industries | 10.3390/microorganisms8030434 |
| 2020-Biochemistry, genetics and biotechnology of glycerol utilization in Pseudomonas species | 10.1111/1751-7915.13400 |
| 2020-Can Polyhydroxyalkanoates Be Produced Efficiently From Waste Plant and Animal Oils? | 10.3389/fbioe.2020.00169 |
| 2020-Cell-based and cell-free biocatalysis for the production of d-glucaric acid | 10.1186/s13068-020-01847-0 |
| 2020-Chapter Three - Clostridium thermocellum: A microbial platform for high-value chemical production from lignocellulose | 10.1016/bs.aambs.2020.07.004 |
| 2020-Current state of aromatics production using yeast: achievements and challenges | 10.1016/j.copbio.2020.01.008 |
| 2020-Diamine Biosynthesis: Research Progress and Application Prospects | 10.1128/aem.01972-20 |
| 2020-Diatoms for Carbon Sequestration and Bio-Based Manufacturing | 10.3390/biology9080217 |
| 2020-Engineering Native and Synthetic Pathways in Pseudomonas putida for the Production of Tailored Polyhydroxyalkanoates | 10.1002/biot.202000165 |
| 2020-Engineering of natural product biosynthesis in Pseudomonas putida | 10.1016/j.copbio.2020.03.007 |
| 2020-Engineering Photosynthetic Bioprocesses for Sustainable Chemical Production: A Review | 10.3389/fbioe.2020.610723 |
| 2020-Metabolic engineering for increased lipid accumulation in Yarrowia lipolytica – A Review | 10.1016/j.biortech.2020.123707 |
| 2020-Metabolic engineering for the production of butanol, a potential advanced biofuel, from renewable resources | 10.1042/bst20200603 |
| 2020-Metabolic engineering for the synthesis of polyesters: A 100-year journey from polyhydroxyalkanoates to non-natural microbial polyesters | 10.1016/j.ymben.2019.05.009 |
| 2020-Metabolic Engineering for Unusual Lipid Production in Yarrowia lipolytica | 10.3390/microorganisms8121937 |
| 2020-Metabolic engineering of Escherichia coli for production of chemicals derived from the shikimate pathway | 10.1007/s10295-020-02288-2 |
| 2020-Metabolic Engineering of Escherichia coli for Natural Product Biosynthesis | 10.1016/j.tibtech.2019.11.007 |
| 2020-Metabolic engineering strategies toward production of biofuels | 10.1016/j.cbpa.2020.02.009 |
| 2020-Metabolic Engineering Escherichia coli for the Production of Lycopene | 10.3390/molecules25143136 |
| 2020-Microbial astaxanthin biosynthesis: recent achievements, challenges, and commercialization outlook | 10.1007/s00253-020-10648-2 |
| 2020-Microbial Polyhydroxyalkanoates and Nonnatural Polyesters | 10.1002/adma.201907138 |
| 2020-Next-generation metabolic engineering of non-conventional microbial cell factories for carboxylic acid platform chemicals | 10.1016/j.biotechadv.2020.107605 |
| 2020-Pentose metabolism and conversion to biofuels and high-value chemicals in yeasts | 10.1093/femsre/fuaa069 |
| 2020-Photosynthetic Conversion of Carbon Dioxide to Oleochemicals by Cyanobacteria: Recent Advances and Future Perspectives | 10.3389/fmicb.2020.00634 |
| 2020-Positioning Bacillus subtilis as terpenoid cell factory | 10.1111/jam.14904 |
| 2020-Production of plant natural products through engineered Yarrowia lipolytica | 10.1016/j.biotechadv.2020.107555 |
| 2020-Production of Terpenoids by Synthetic Biology Approaches | 10.3389/fbioe.2020.00347 |
| 2020-Production of Vitamin B2 (Riboflavin) by Microorganisms: An Overview | 10.3389/fbioe.2020.570828 |
| 2020-Pseudomonas spp. as cell factories (MCFs) for value-added products: from rational design to industrial applications | 10.1080/07388551.2020.1809990 |
| 2020-Pseudomonas as Versatile Aromatics Cell Factory | 10.1002/biot.201900569 |
| 2020-Recent advances in domesticating non-model microorganisms | 10.1002/btpr.3008 |
| 2020-Recent Advances in Microbial Production of cis,cis-Muconic Acid | 10.3390/biom10091238 |
| 2020-Recent advances in modular co-culture engineering for synthesis of natural products | 10.1016/j.copbio.2019.09.004 |
| 2020-Recent Advances in the Metabolic Engineering of Yeasts for Ginsenoside Biosynthesis | 10.3389/fbioe.2020.00139 |
| 2020-Recent advances in n-butanol and butyrate production using engineered Clostridium tyrobutyricum | 10.1007/s11274-020-02914-2 |
| 2020-Recent progress on bio-based production of dicarboxylic acids in yeast | 10.1007/s00253-020-10537-8 |
| 2020-Recent Progress on Chemical Production From Non-food Renewable Feedstocks Using Corynebacterium glutamicum | 10.3389/fbioe.2020.606047 |
| 2020-Revisiting metabolic engineering strategies for microbial synthesis of oleochemicals | 10.1016/j.ymben.2019.04.009 |
| 2020-Synthetic Biology and Metabolic Engineering Employing Escherichia coli for C2-C6 Bioalcohol Production | 10.3389/fbioe.2020.00710 |
| 2020-Synthetic Biology Approaches to Engineer Saccharomyces cerevisiae towards the Industrial Production of Valuable Polyphenolic Compounds | 10.3390/life10050056 |
| 2020-Synthetic biology, systems biology, and metabolic engineering of Yarrowia lipolytica toward a sustainable biorefinery platform | 10.1007/s10295-020-02290-8 |
| 2020-The Application of Regulatory Cascades in Streptomyces: Yield Enhancement and Metabolite Mining | 10.3389/fmicb.2020.00406 |
| 2020-The Gibberellin Producer Fusarium fujikuroi: Methods and Technologies in the Current Toolkit | 10.3389/fbioe.2020.00232 |
| 2020-Towards continuous industrial bioprocessing with solventogenic and acetogenic clostridia: challenges, progress and perspectives | 10.1007/s10295-020-02296-2 |
| 2020-Valorisation of pectin-rich agro-industrial residues by yeasts: potential and challenges | 10.1007/s00253-020-10697-7 |
| 2020-Yarrowia lipolytica as an Oleaginous Platform for the Production of Value-Added Fatty Acid-Based Bioproducts | 10.3389/fmicb.2020.608662 |
| 2020-Yeast metabolic engineering for the production of pharmaceutically important secondary metabolites | 10.1007/s00253-020-10587-y |
| 2021-A bioprocess perspective on the production of secondary metabolites by Streptomyces in submerged co-cultures | 10.1007/s11274-021-03141-z |
| 2021-A Review of the Recent Developments in the Bioproduction of Polylactic Acid and Its Precursors Optically Pure Lactic Acids | 10.3390/molecules26216446 |
| 2021-Advanced Strategies for the Synthesis of Terpenoids in Yarrowia lipolytica | 10.1021/acs.jafc.1c00350 |
| 2021-Approaches in the photosynthetic production of sustainable fuels by cyanobacteria using tools of synthetic biology | 10.1007/s11274-021-03157-5 |
| 2021-Bioconversion of Lignocellulosic Biomass into Value Added Products under Anaerobic Conditions: Insight into Proteomic Studies | 10.3390/ijms222212249 |
| 2021-Biodiesel Production From Lignocellulosic Biomass Using Oleaginous Microbes: Prospects for Integrated Biofuel Production | 10.3389/fmicb.2021.658284 |
| 2021-Bioproduction process of natural products and biopharmaceuticals: Biotechnological aspects | 10.1016/j.biotechadv.2021.107768 |
| 2021-Biosynthesis and applications of curdlan | 10.1016/j.carbpol.2021.118597 |
| 2021-Biosynthesis pathways and strategies for improving 3-hydroxypropionic acid production in bacteria | 10.1007/s11274-021-03091-6 |
| 2021-Biotechnological applications of the non-conventional yeast Meyerozyma guilliermondii | 10.1016/j.biotechadv.2020.107674 |
| 2021-Biotechnological production of lipid and terpenoid from thraustochytrids | 10.1016/j.biotechadv.2021.107725 |
| 2021-Clavulanic Acid Production by Streptomyces clavuligerus: Insights from Systems Biology, Strain Engineering, and Downstream Processing | 10.3390/antibiotics10010084 |
| 2021-Construction of recombinant Escherichia coli for production of l-phenylalanine-derived compounds | 10.1007/s11274-021-03050-1 |
| 2021-Current Advances towards 4-Hydroxybutyrate Containing Polyhydroxyalkanoates Production for Biomedical Applications | 10.3390/molecules26237244 |
| 2021-Depolymerization and conversion of lignin to value-added bioproducts by microbial and enzymatic catalysis | 10.1186/s13068-021-01934-w |
| 2021-Developments in Fatty Acid-Derived Insect Pheromone Production Using Engineered Yeasts | 10.3389/fmicb.2021.759975 |
| 2021-Diversifying Isoprenoid Platforms via Atypical Carbon Substrates and Non-model Microorganisms | 10.3389/fmicb.2021.791089 |
| 2021-Endophytes, biotransforming microorganisms, and engineering microbial factories for triterpenoid saponins production | 10.1080/07388551.2020.1869691 |
| 2021-Engineering of microbial cells for L-valine production: challenges and opportunities | 10.1186/s12934-021-01665-5 |
| 2021-Engineering of xylose metabolism in Escherichia coli for the production of valuable compounds | 10.1080/07388551.2021.1873243 |
| 2021-Engineering xylose metabolism in yeasts to produce biofuels and chemicals | 10.1016/j.copbio.2020.10.012 |
| 2021-Engineering Yarrowia lipolytica to produce advanced biofuels: Current status and perspectives | 10.1016/j.biortech.2021.125877 |
| 2021-Engineering Yarrowia lipolytica to produce fuels and chemicals from xylose: A review | 10.1016/j.biortech.2021.125484 |
| 2021-Engineering Saccharomyces cerevisiae to produce plant benzylisoquinoline alkaloids | 10.1007/s42994-021-00055-0 |
| 2021-From Residues to Added-Value Bacterial Biopolymers as Nanomaterials for Biomedical Applications | 10.3390/nano11061492 |
| 2021-Genome-Wide Metabolic Reconstruction of the Synthesis of Polyhydroxyalkanoates from Sugars and Fatty Acids by Burkholderia Sensu Lato Species | 10.3390/microorganisms9061290 |
| 2021-Halomonas as a chassis | 10.1042/ebc20200159 |
| 2021-Harnessing the yeast Saccharomyces cerevisiae for the production of fungal secondary metabolites | 10.1042/ebc20200137 |
| 2021-l-arginine production in Corynebacterium glutamicum: manipulation and optimization of the metabolic process | 10.1080/07388551.2020.1844625 |
| 2021-Lignin valorization by bacterial genus Pseudomonas: State-of-the-art review and prospects | 10.1016/j.biortech.2020.124412 |
| 2021-L-valine production in Corynebacterium glutamicum based on systematic metabolic engineering: progress and prospects | 10.1007/s00726-021-03066-9 |
| 2021-Metabolic engineering and synthetic biology for isoprenoid production in Escherichia coli and Saccharomyces cerevisiae | 10.1007/s00253-020-11040-w |
| 2021-Metabolic engineering for high yield synthesis of astaxanthin in Xanthophyllomyces dendrorhous | 10.1186/s12934-021-01664-6 |
| 2021-Metabolic engineering of Escherichia coli for the production of isobutanol: a review | 10.1007/s11274-021-03140-0 |
| 2021-Metabolic Engineering of Microbial Cell Factories for Biosynthesis of Flavonoids: A Review | 10.3390/molecules26154522 |
| 2021-Metabolic engineering of microorganisms for L-alanine production | 10.1093/jimb/kuab057 |
| 2021-Metabolic engineering of non-pathogenic microorganisms for 2,3-butanediol production | 10.1007/s00253-021-11436-2 |
| 2021-Metabolic engineering of Yarrowia lipolytica for terpenoids production: advances and perspectives | 10.1080/07388551.2021.1947183 |
| 2021-Metabolic engineering of Corynebacterium glutamicum for producing branched chain amino acids | 10.1186/s12934-021-01721-0 |
| 2021-Metabolic engineering of Saccharomyces cerevisiae for the production of top value chemicals from biorefinery carbohydrates | 10.1016/j.biotechadv.2021.107697 |
| 2021-Metabolic engineering strategy for synthetizing trans-4-hydroxy-l-proline in microorganisms | 10.1186/s12934-021-01579-2 |
| 2021-Microbial and Genetic Resources for Cobalamin (Vitamin B12) Biosynthesis: From Ecosystems to Industrial Biotechnology | 10.3390/ijms22094522 |
| 2021-Microbial application of thermophilic Thermoanaerobacterium species in lignocellulosic biorefinery | 10.1007/s00253-021-11450-4 |
| 2021-Microbial biotechnological approaches: renewable bioprocessing for the future energy systems | 10.1186/s12934-021-01547-w |
| 2021-Microbial production of advanced biofuels | 10.1038/s41579-021-00577-w |
| 2021-Microbial production of ectoine and hydroxyectoine as high-value chemicals | 10.1186/s12934-021-01567-6 |
| 2021-Microbial production of gamma-aminobutyric acid: applications, state-of-the-art achievements, and future perspectives | 10.1080/07388551.2020.1869688 |
| 2021-Microbial production of riboflavin: Biotechnological advances and perspectives | 10.1016/j.ymben.2021.08.009 |
| 2021-New aspects of microbial vitamin K2 production by expanding the product spectrum | 10.1186/s12934-021-01574-7 |
| 2021-Physiological characteristics of Corynebacterium glutamicum as a cell factory under anaerobic conditions | 10.1007/s00253-021-11474-w |
| 2021-Production of l-glutamate family amino acids in Corynebacterium glutamicum: Physiological mechanism, genetic modulation, and prospects | 10.1016/j.synbio.2021.09.005 |
| 2021-Promising advancement in fermentative succinic acid production by yeast hosts | 10.1016/j.jhazmat.2020.123414 |
| 2021-Recent advances in constructing artificial microbial consortia for the production of medium-chain-length polyhydroxyalkanoates | 10.1007/s11274-020-02986-0 |
| 2021-Recent Advances in Heterologous Synthesis Paving Way for Future Green-Modular Bioindustries: A Review With Special Reference to Isoflavonoids | 10.3389/fbioe.2021.673270 |
| 2021-Recent Advances in Lactic Acid Production by Lactic Acid Bacteria | 10.1007/s12010-021-03672-z |
| 2021-Recent advances in lipid metabolic engineering of oleaginous yeasts | 10.1016/j.biotechadv.2021.107722 |
| 2021-Recent Advances in Synthetic, Industrial and Biological Applications of Violacein and Its Heterologous Production | 10.4014/jmb.2107.07045 |
| 2021-Recent advances in systems and synthetic biology approaches for developing novel cell-factories in non-conventional yeasts | 10.1016/j.biotechadv.2021.107695 |
| 2021-Recent advances in the biological valorization of citrus peel waste into fuels and chemicals | 10.1016/j.biortech.2020.124603 |
| 2021-Recent progress in production of amino acid‐derived chemicals using Corynebacterium glutamicum | 10.1007/s11274-021-03007-4 |
| 2021-Research advances on arachidonic acid production by fermentation and genetic modification of Mortierella alpina | 10.1007/s11274-020-02984-2 |
| 2021-Resveratrol Production in Yeast Hosts: Current Status and Perspectives | 10.3390/biom11060830 |
| 2021-Rhodotorula toruloides: an ideal microbial cell factory to produce oleochemicals, carotenoids, and other products | 10.1007/s11274-021-03201-4 |
| 2021-Saccharomyces cerevisiae as host for the recombinant production of polyketides and nonribosomal peptides | 10.1186/s12934-021-01650-y |
| 2021-Strategies to Increase the Production of Biosynthetic Riboflavin | 10.1007/s12033-021-00318-7 |
| 2021-Streptomycetes as platform for biotechnological production processes of drugs | 10.1007/s00253-020-11064-2 |
| 2021-Successful Enzyme Colocalization Strategies in Yeast for Increased Synthesis of Non-native Products | 10.3389/fbioe.2021.606795 |
| 2021-Synthetic biology toolkit for engineering Cupriviadus necator H16 as a platform for CO2 valorization | 10.1186/s13068-021-02063-0 |
| 2021-The aminoshikimic acid pathway in bacteria as source of precursors for the synthesis of antibacterial and antiviral compounds | 10.1093/jimb/kuab053 |
| 2021-Understanding D-xylonic acid accumulation: a cornerstone for better metabolic engineering approaches | 10.1007/s00253-021-11410-y |
| 2021-Using Co-Culture to Functionalize Clostridium Fermentation | 10.1016/j.tibtech.2020.11.016 |
| 2021-Yarrowia lipolytica as an emerging biotechnological chassis for functional sugars biosynthesis | 10.1080/10408398.2020.1739000 |
| 2021-Yeast-Based Biosynthesis of Natural Products From Xylose | 10.3389/fbioe.2021.634919 |
| 2021-Yeasts of the Blastobotrys genus are promising platform for lipid-based fuels and oleochemicals production | 10.1007/s00253-021-11354-3 |
| 2022-A Review on Enhancing Cupriavidus necator Fermentation for Poly(3-hydroxybutyrate) (PHB) Production From Low-Cost Carbon Sources | 10.3389/fbioe.2022.946085 |
| 2022-Advances and prospects in metabolic engineering of Escherichia coli for L-tryptophan production | 10.1007/s11274-021-03212-1 |
| 2022-Advances and trends in microbial production of polyhydroxyalkanoates and their building blocks | 10.3389/fbioe.2022.966598 |
| 2022-Advances in synthetic biology tools paving the way for the biomanufacturing of unusual fatty acids using the Yarrowia lipolytica chassis | 10.1016/j.biotechadv.2022.107984 |
| 2022-Advances in systems metabolic engineering of autotrophic carbon oxide-fixing biocatalysts towards a circular economy | 10.1016/j.ymben.2022.01.015 |
| 2022-Alternative metabolic pathways and strategies to high-titre terpenoid production in Escherichia coli | 10.1039/D1NP00025J |
| 2022-An updated review on advancement in fermentative production strategies for biobutanol using Clostridium spp | 10.1007/s11356-022-20637-9 |
| 2022-Bacillus licheniformis: The unexplored alternative for the anaerobic production of lipopeptide biosurfactants? | 10.1016/j.biotechadv.2022.108013 |
| 2022-Bacterial conversion routes for lignin valorization | 10.1016/j.biotechadv.2022.108000 |
| 2022-Bioengineering for the industrial production of 2,3-butanediol by the yeast, Saccharomyces cerevisiae | 10.1007/s11274-021-03224-x |
| 2022-Bioprospecting Kluyveromyces marxianus as a Robust Host for Industrial Biotechnology | 10.3389/fbioe.2022.851768 |
| 2022-Biotechnological production and application of epsilon-poly-L-lysine (ε-PL): biosynthesis and its metabolic regulation | 10.1007/s11274-022-03304-6 |
| 2022-Caproic Acid-Producing Bacteria in Chinese Baijiu Brewing | 10.3389/fmicb.2022.883142 |
| 2022-Current status, challenges and prospects for lignin valorization by using Rhodococcus sp | 10.1016/j.biotechadv.2022.108004 |
| 2022-Efficient cell factories for the production of N‐methylated amino acids and for methanol‐based amino acid production | 10.1111/1751-7915.14067 |
| 2022-Engineered bacteria for valorizing lignocellulosic biomass into bioethanol | 10.1016/j.biortech.2021.126212 |
| 2022-Engineering E. coli to synthesize butanol | 10.1042/bst20211009 |
| 2022-Engineering Yarrowia lipolytica to produce nutritional fatty acids: Current status and future perspectives | 10.1016/j.synbio.2022.06.002 |
| 2022-Enhancing microbial lipids yield for biodiesel production by oleaginous yeast Lipomyces starkeyi fermentation: A review | 10.1016/j.biortech.2021.126294 |
| 2022-Feedback regulation and coordination of the main metabolism for bacterial growth and metabolic engineering for amino acid fermentation | 10.1016/j.biotechadv.2021.107887 |
| 2022-Glutathione production by Saccharomyces cerevisiae: current state and perspectives | 10.1007/s00253-022-11826-0 |
| 2022-Heterologous production of chondroitin | 10.1016/j.btre.2022.e00710 |
| 2022-Industrial production of L-lysine in Corynebacterium glutamicum: Progress and prospects | 10.1016/j.micres.2022.127101 |
| 2022-Insights into cyanobacterial alkane biosynthesis | 10.1093/jimb/kuab075 |
| 2022-Leads and hurdles to sustainable microbial bioplastic production | 10.1016/j.chemosphere.2022.135390 |
| 2022-Mannitol Production by Heterofermentative Lactic Acid Bacteria: a Review | 10.1007/s12010-022-03836-5 |
| 2022-Metabolic Engineering and Regulation of Diol Biosynthesis from Renewable Biomass in Escherichia coli | 10.3390/biom12050715 |
| 2022-Metabolic Engineering for Valorization of Agri- and Aqua-Culture Sidestreams for Production of Nitrogenous Compounds by Corynebacterium glutamicum | 10.3389/fmicb.2022.835131 |
| 2022-Metabolic Engineering of Shikimic Acid Biosynthesis Pathway for the Production of Shikimic Acid and Its Branched Products in Microorganisms: Advances and Prospects | 10.3390/molecules27154779 |
| 2022-Metabolic engineering strategies for sesquiterpene production in microorganism | 10.1080/07388551.2021.1924112 |
| 2022-MICROBIAL isoprene production: an overview | 10.1007/s11274-022-03306-4 |
| 2022-Microbial production of lactic acid from food waste: Latest advances, limits, and perspectives | 10.1016/j.biortech.2021.126052 |
| 2022-Microbial Utilization of Next-Generation Feedstocks for the Biomanufacturing of Value-Added Chemicals and Food Ingredients | 10.3389/fbioe.2022.874612 |
| 2022-Mucor circinelloides: a model organism for oleaginous fungi and its potential applications in bioactive lipid production | 10.1186/s12934-022-01758-9 |
| 2022-Optimization of microbial cell factories for astaxanthin production: Biosynthesis and regulations, engineering strategies and fermentation optimization strategies | 10.1016/j.synbio.2022.01.002 |
| 2022-Production, Biosynthesis, and Commercial Applications of Fatty Acids From Oleaginous Fungi | 10.3389/fnut.2022.873657 |
| 2022-Recent advances in construction and regulation of yeast cell factories | 10.1007/s11274-022-03241-4 |
| 2022-Recent advances in metabolic regulation and bioengineering of gibberellic acid biosynthesis in Fusarium fujikuroi | 10.1007/s11274-022-03324-2 |
| 2022-Recent Advances in Microbial Synthesis of Poly-γ-Glutamic Acid: A Review | 10.3390/foods11050739 |
| 2022-Recent advances in the metabolic pathways and microbial production of coenzyme Q | 10.1007/s11274-022-03242-3 |
| 2022-Recent advances in the microbial production of squalene | 10.1007/s11274-022-03273-w |
| 2022-Recent progress in strategies for steroid production in yeasts | 10.1007/s11274-022-03276-7 |
| 2022-Recent Progress on Systems and Synthetic Biology of Diatoms for Improving Algal Productivity | 10.3389/fbioe.2022.908804 |
| 2022-Response mechanisms of Saccharomyces cerevisiae to the stress factors present in lignocellulose hydrolysate and strategies for constructing robust strains | 10.1186/s13068-022-02127-9 |
| 2022-The industrial versatility of Gluconobacter oxydans: current applications and future perspectives | 10.1007/s11274-022-03310-8 |
| 2022-The soil bacterium, Corynebacterium glutamicum, from biosynthesis of value-added products to bioremediation: A master of many trades | 10.1016/j.envres.2022.113622 |
