# Supplementary material for: MCF2Chem: A manually curated knowledge base of biosynthetic compound production
Source: Biotechnol Biofuels Bioprod. 2023 Nov 4;16:167. doi: 10.1186/s13068-023-02419-8 (PMC10625697; doi:10.1186/s13068-023-02419-8)
Supplement: Supplementary file 2 — Additional file 2: Figure S1. Top 10 journals contributing the most reviews used for data extraction. Figure S2. Time statistics of the original articles, countries, and institutions for microbial cell factory biosynthesis. Figure S3. Time statistics of the top 20 journals contributing the most original articles on microbial cell factory biosynthesis. Figure S4. Development timeline of the average titer of microbial cell factory biosynthesis. Figure S5. Time statistics of the average titer of microbial cell factory biosynthesis in every product category. Figure S6. Global distribution of microbial cell factory biosynthetic chemical products. Figure S7. Top 10 countries contributing the most data to microbial cell factory biosynthesis. Figure S8. Top 10 institutions contributing the most data to microbial cell factory biosynthesis. Figure S9. Timeline depicting trends in the development of various aspects of microbial cell factory biosynthesis. Table S2. MCF2Chem database coverage statistical analysis using the journal Metabolic Engineering as an example. [file 13068_2023_2419_MOESM2_ESM.docx]

Fig. S1 Top 10 journals contributing the most reviews used for data extraction.

Fig. S2 Time statistics of the original articles, countries, and institutions for microbial cell factory biosynthesis.


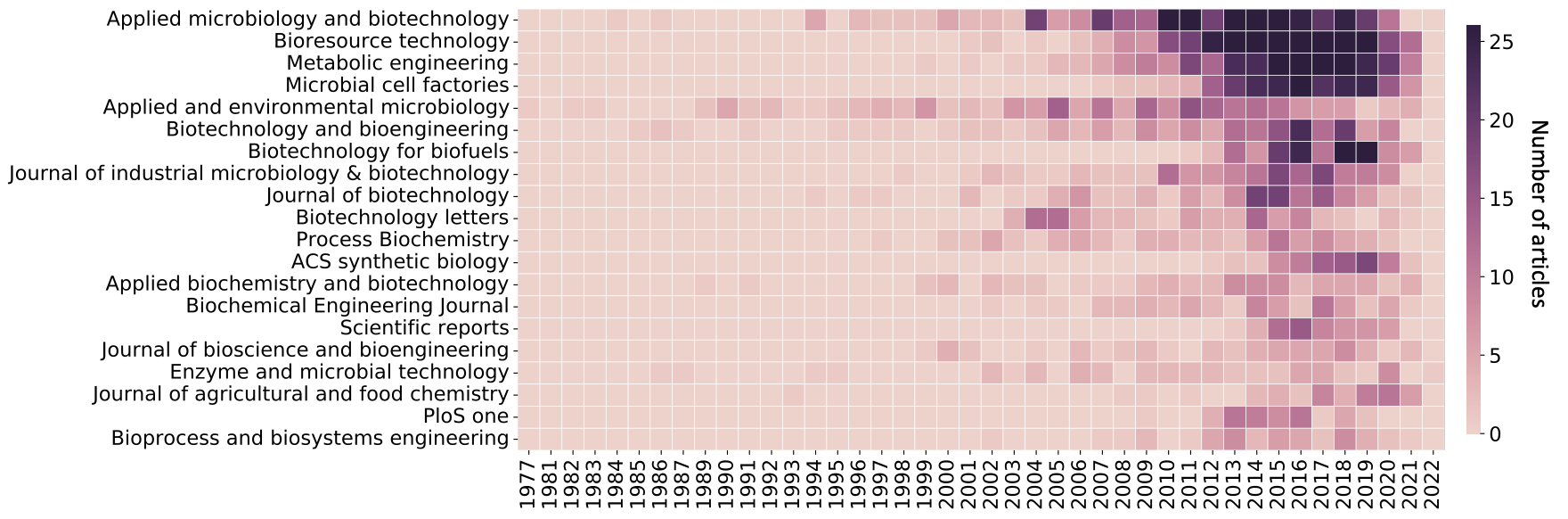


Fig. S3 Time statistics of the top 20 journals contributing the most original articles on microbial cell factory biosynthesis.


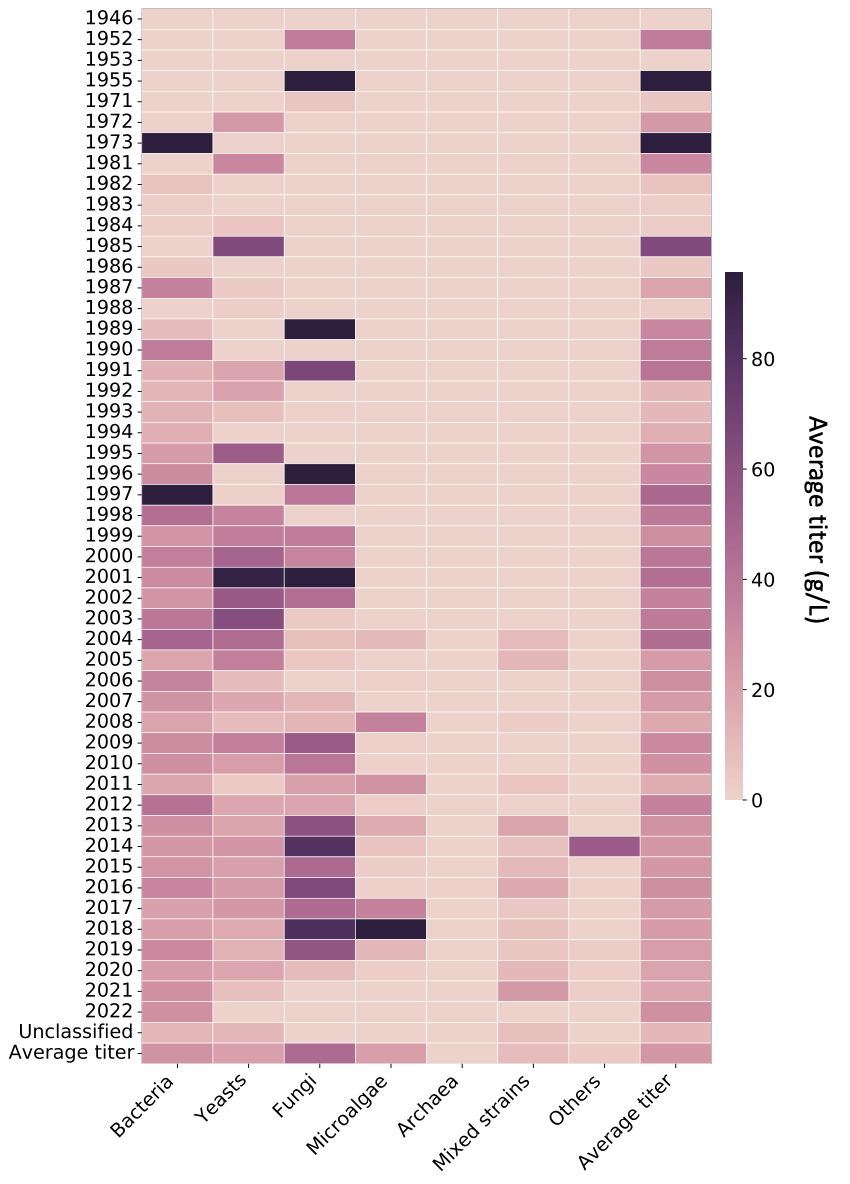


Fig. S4 Development timeline of the average titer of microbial cell factory biosynthesis.


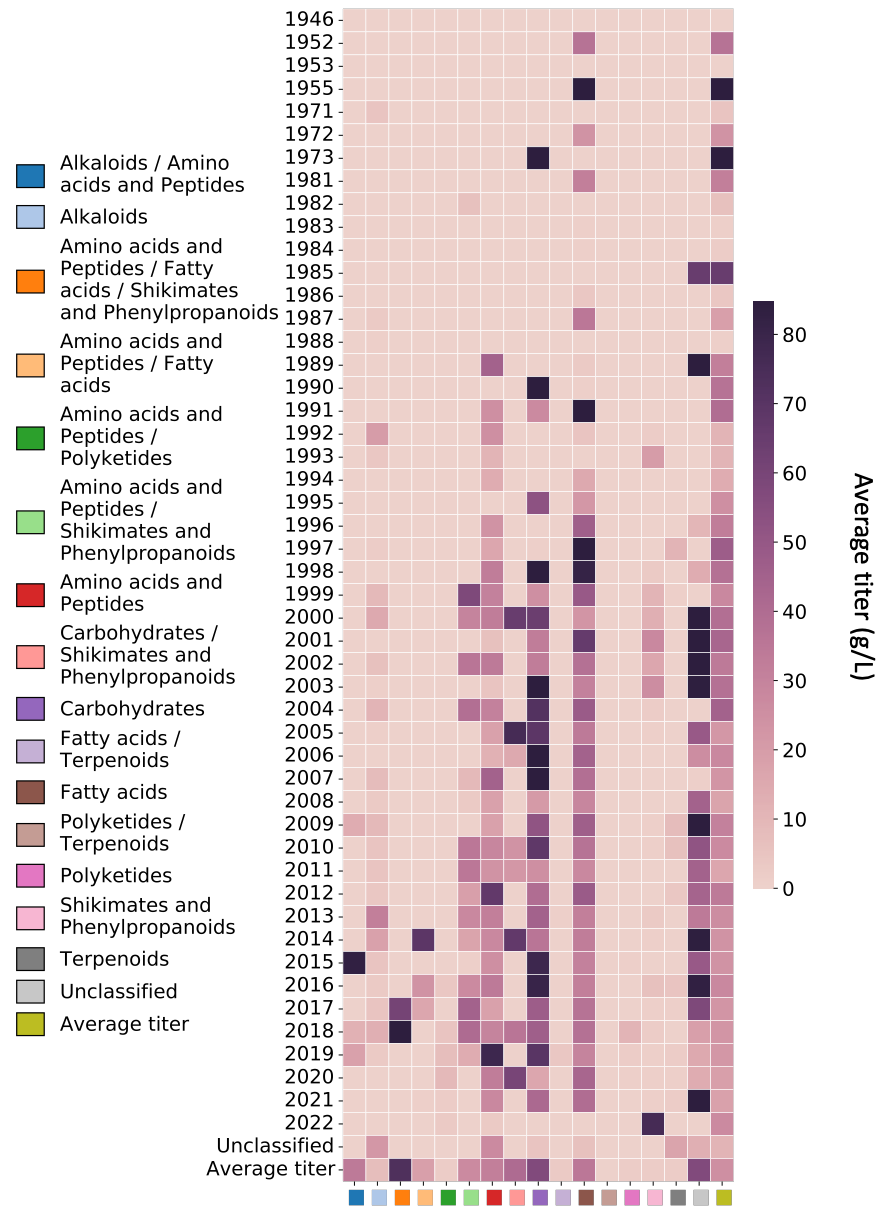


Fig. S5 Time statistics of the average titer of microbial cell factory biosynthesis in every product category.


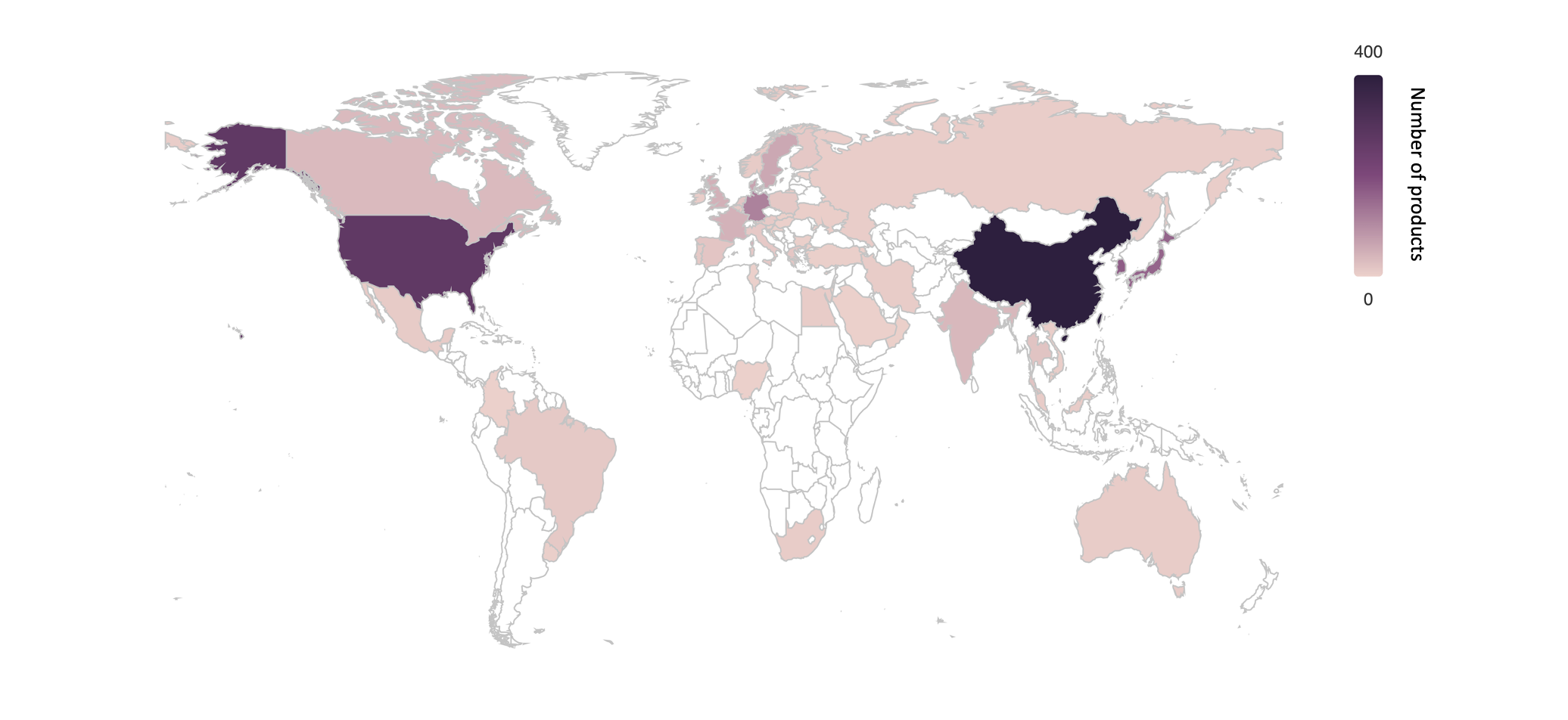


Fig. S6 Global distribution of microbial cell factory biosynthetic chemical products.

Fig. S7 Top 10 countries contributing the most data to microbial cell factory biosynthesis.

Fig. S8 Top 10 institutions contributing the most data to microbial cell factory biosynthesis.

Fig. S9 Timeline depicting trends in the development of various aspects of microbial cell factory biosynthesis.

Table S2 MCF2Chem database coverage statistical analysis using the journal *Metabolic Engineering* as an example.

| Year | 2016 | 2017 | 2018 |
| --- | --- | --- | --- |
| Number of articles collected by MCF2Chem | 40 | 42 | 36 |
| Number of articles published in Metabolic Engineering | 61 | 65 | 60 |
| MCF2Chem coverage rate | 66% | 65% | 60% |
| MCF2Chem average coverage rate | 63% | | |
