# Supplementary material for: MCF2Chem: A manually curated knowledge base of biosynthetic compound production
Source: Biotechnol Biofuels Bioprod. 2023 Nov 4;16:167. doi: 10.1186/s13068-023-02419-8 (PMC10625697; doi:10.1186/s13068-023-02419-8)
Supplement: Supplementary file 3 — Additional file 3. Scoring functions for chemical and species recommendation. [file 13068_2023_2419_MOESM3_ESM.docx]

**Scoring functions for chemical and species recommendation**

$$\begin{aligned} \boldsymbol{rc=lo}\boldsymbol{g}_{\boldsymbol{3}}\left( \boldsymbol{p+}\frac{\boldsymbol{w}_{\boldsymbol{1}}\boldsymbol{t+}\boldsymbol{w}_{\boldsymbol{2}}\boldsymbol{n}}{\boldsymbol{w}_{\boldsymbol{1}}\boldsymbol{+}\boldsymbol{w}_{\boldsymbol{2}}}\boldsymbol{+1} \right)\boldsymbol{\#}\boldsymbol{(}\boldsymbol{1}\boldsymbol{)} \end{aligned}$$

$\boldsymbol{rc}$ indicates the recommended score of one compound;

$\boldsymbol{t}$ is the normalized titer of one compound, normalization method is min-max;

$\boldsymbol{n}$ is the normalized production record count of one compound, normalization method is min-max;

$\boldsymbol{w}_{\boldsymbol{1}}$ and $\boldsymbol{w}_{\boldsymbol{2}}$ denote different weighting factors, here they are 0.7 and 0.2 respectively;

$$\begin{aligned} \boldsymbol{rs=lo}\boldsymbol{g}_{\boldsymbol{3}}\left( \boldsymbol{p+}\frac{\boldsymbol{w}_{\boldsymbol{1}}\boldsymbol{t+}\boldsymbol{w}_{\boldsymbol{2}}\boldsymbol{n+}\boldsymbol{w}_{\boldsymbol{3}}\boldsymbol{c+}\boldsymbol{w}_{\boldsymbol{4}}\boldsymbol{g+}\boldsymbol{w}_{\boldsymbol{5}}\boldsymbol{s+}\boldsymbol{w}_{\boldsymbol{6}}\boldsymbol{m}}{\boldsymbol{w}_{\boldsymbol{1}}\boldsymbol{+}\boldsymbol{w}_{\boldsymbol{2}}\boldsymbol{+}\boldsymbol{w}_{\boldsymbol{3}}\boldsymbol{+}\boldsymbol{w}_{\boldsymbol{4}}\boldsymbol{+}\boldsymbol{w}_{\boldsymbol{5}}\boldsymbol{+}\boldsymbol{w}_{\boldsymbol{6}}}\boldsymbol{+1} \right)\boldsymbol{\#}\boldsymbol{(}\boldsymbol{2}\boldsymbol{)} \end{aligned}$$

$\boldsymbol{rs}$ indicates the recommended score of one species;

$\boldsymbol{t}$ is the normalized mean titer for one species, normalization method is min-max;

$\boldsymbol{n}$ is the normalized production record count for one species, normalization method is min-max;

$\boldsymbol{c}$, $\boldsymbol{g}$, $\boldsymbol{s}$, $\boldsymbol{m}$ represent the presence or absence of culture media, genetic operating system, genome sequencing and genomic metabolic network model for one species respectively, 1 if yes, 0 if no; $\boldsymbol{w}_{\boldsymbol{1}}$, $\boldsymbol{w}_{\boldsymbol{2}}$**,** $\boldsymbol{w}_{\boldsymbol{3}}$, $\boldsymbol{w}_{\boldsymbol{4}}$, $\boldsymbol{w}_{\boldsymbol{5}}$ and $\boldsymbol{w}_{\boldsymbol{6}}$ denote different weighting factors, here they are 0.7, 0.2, 0.1, 0.1, 0.1 and 0.1 respectively;

$\boldsymbol{p}$ is the recommendation route score, calculation varies according to the different routes:

| **Route** | **Score** |
| --- | --- |
| S2C | 1 |
| S2C2C | 1* sim (c1, c2) |
| S2S2C | 1/ (1 + dist (s1, s2)) *1 |
| C2S | 1 |
| C2S2S | 1*1/ (1 + dist (s1, s2)) |
| C2C2S | sim (c1, c2) *1 |

where sim (c1, c2) represents the structural similarity of the two compounds and dist (s1, s2) represents the evolutionary distance between the two species.
